# Virtual reconstruction and analysis of the face of DFN3-150 *Paradolichopithecus* aff. *arvernensis* specimen from Dafnero, Greece

**DOI:** 10.1038/s41598-026-51595-8

**Published:** 2026-05-10

**Authors:** Stylianos Koutalis, Carolin Röding, Gildas Merceron, Franck Guy, Dimitris S. Kostopoulos, Katerina Harvati

**Affiliations:** 1 Paleoanthropology, Department of Geosciences, Senckenberg Centre for Human Evolution and Palaeoenvironment, Eberhard Karls University of Tübingen, Tübingen, Germany; 2 Paleoanthropology, Institute for Archaeological Sciences, Eberhard Karls University of Tübingen, Rümelinstr. 23, 72070 Tübingen, Germany; 3 Laboratoire PALEVOPRIM, UMR 7262 CNRS & University of Poitiers, 6 rue Michel Brunet, 86073 Poitiers Cedex 9, France; 4 School of Geology, Aristotle University of Thessaloniki, 54124 Thessaloniki, Greece; 5 DFG Center for Advanced Studies ‘Words, Bones, Genes, Tools: Tracking Linguistic, Cultural and Biological Trajectories of the Human Past’, Eberhard Karls University of Tübingen, Tübingen, Germany; 6 Human Origins – Cluster of Excellence for Integrative Human Origins Studies (EXC 3101), Eberhard Karls University of Tübingen, Rümelinstr. 23, 72070 Tübingen, Germany

**Keywords:** Cercopithecinae, Villafranchian, Retrodeformation, Allometry, Modeling, Ecology, Ecology, Evolution, Zoology

## Abstract

The systematics of the Eurasian Late Pliocene – Early Pleistocene monkey *Paradolichopithecus* is a long-debated topic in primate paleontology. This taxon’s phylogenetic position has implications for our understanding of cercopithecid evolution: while it is often regarded as a baboon-like Macacina, an alternative taxonomic attribution to the Papionina subtribe has been proposed. This would challenge the commonly accepted hypothesis that baboons constitute an endemic African clade. Here, we reconstructed the face of the subadult female DFN3-150 *Paradolichopithecus* aff. *arvernensis* cranium from the Lower Pleistocene site Dafnero (Greece), using novel virtual techniques and following alternative protocols. We performed a geometric morphometrics investigation of its morphological affinities with two extant papionin genera. Our results allow for a critical evaluation of the two applied retrodeformation protocols and support stronger facial morphological similarities with *Papio* spp. than macaques. This is in agreement with recent studies evaluating its phenotypic and ecological traits. Nevertheless, direct inferences on phylogenetic affinities require a larger and more diverse papionin sample.

**Supplementary Information:**

The online version contains supplementary material available at 10.1038/s41598-026-51595-8.

## Introduction

The subfamily Cercopithecinae diverged as a clade of Cercopithecidae around the Early to Middle Miocene (14.4–20.6 Ma)^1–6^. It is divided into two tribes, Cercopithecini and Papionini, the latter of which further diversified in the Late Miocene (approximately between 8 and 11 Ma) into the subtribes Macacina (*Macaca* lineage) and Papionina (baboons and relatives/*Papio* lineage)^2,3,6^. The resolution of their phylogeny is complicated by a strong evolutionary allometric component that affects cranial shape variation and thus confuses taxonomic indicators based on overall shape, possibly due to high levels of parallel size evolution^7–9^. The Eurasian *Paradolichopithecus* (Depéret 1929), together with its synchronous and possibly synonymous taxon, *Procynocephalus* Schlosser, 1924^10–13^, occupy a crucial position in the discussion of cercopithecid systematics.

*Paradolichopithecus* is commonly viewed as a member of the Macacina^12,14^, but an alternative phylogeny closer or within Papionina has also been proposed^15,16^. Depending on the still unknown resolution of the phylogenetic relationships, such an attribution would challenge the general consensus that *Papio* spp. are an African endemic group.

*Paradolichopithecus* is the largest cercopithecine in the Eurasian fossil record. It covers a timespan from the middle Pliocene to the Early Pleistocene and four species are typically recognized. European forms are assigned to *Paradolichopithecus arvernensis* (Depéret, 1929), and *Paradolichopithecus geticus* Necrasov, Samson, and Radulesco, 1961, which are also suggested as possible synonyms^17,18^. *Paradolichopithecus sushkini* Trofimov, 1977 is known from central Asia and *Paradolichopithecus gansuensis* Qui, Deng and Wang, 2004 from China. Together, these taxa cover the entire latitudinal sub-Alpine belt from Spain to China^10,19–22^.

Cranially, these large-bodied cercopithecines are reported to resemble macaques^10–12,17,19,23^, while postcranially they are more similar to baboons (*Papio* and *Theropithecus*) in that they display features associated with dedicated terrestrial quadrupedalism^10,12,24^. Cranial characters of *Paradolichopithecus*, reported to place the genus closer to the *Macaca* lineage^10^, include a gently concave frontonasal profile, broad palate, a small maxillary sinus, weak to absent maxillary fossae, as well as a thick maxillary body. The presence of a maxillary sinus especially is a key element to attribute fossil specimens into Macacina. However, not all *Paradolichopithecus* specimens present a maxillary sinus^25^. Furthermore, a maxillary sinus is also found in some *Papio* and *Theropithecus* specimens^13^. On the other hand, the absence of maxillary fossae is also observed in primitive papionins^26^ and could be a primitive retention in *Paradolichopithecus*. *Papio*-like cranial features traced on *Paradolichopithecus*, like the complete engulfment of the lacrimal fossa in the lacrimal bone, are expected to be less stereotypical in the fossil taxon, due to its primitive state^10,11,13^, and therefore of more limited systematic value^10,11,13^. In the case of *Paradolichopithecus sushkini*, however, the reported presence of a deep mandibular and pronounced maxillary fossae, along with a lacrimal fossa located completely in the lacrimal bone have been proposed to indicate direct phylogenetic proximity to baboons^15,16^.

DFN3-150 is one of the few complete *Paradolichopithecus* crania known^10^. As such, it is an important specimen for testing hypotheses about this species’ phylogenetic position within the Papionini clade. Previous work^10^ has shown that it shares a maxillary sinus with macaques, however the phylogenetic significance of this trait has been questioned^10,13^. Furthermore, a geometric morphometric investigation of its inner ear morphology^27^ suggested a morphology more consistent with a stem Papionini closer to Papionina than Macacina, or to a basal crown Papionina. These findings contrast with studies of other *Paradolichopithecus* specimens, conducted with both traditional and geometric morphometrics approaches, which suggest a closer relationship to macaques^14^, despite their larger size and associated terrestrial locomotor adaptations^28,29^.

Size variation is particularly pronounced among Papionini^7,9,30–33^, and their morphology is strongly affected by allometric relationships. Therefore, observed similarities of *Paradolichopithecus* with baboons could be a matter of parallel evolution related to similar body and cranial size^27,34^. Further complicating its interpretation, DFN3-150 suffers from both fragmentation and plastic deformation, the latter mainly affecting its right side^10^. While this is clearly visible on the specimen, the differential nature of deformation, namely which side is intact, has not been investigated and quantified^35^.

DFN3-150 cranium is part of the middle Villafranchian fauna recovered at the Lower Pleistocene fossiliferous site Dafnero (NW Greece)^10,36–38^, by a joint research team from the Laboratory of Geology and Paleontology, Aristotle University of Thessaloniki and the PALEVOPRIM Lab, CNRS and University of Poitiers. The specimen is housed at the Museum of Geology-Paleontology-Paleoanthropology (LGPUT), Aristotle University of Thessaloniki. Recognized as a subadult female individual (dental score 4)^10^, based on the presence of erupting adult third molars^39^, it is attributed to *Paradolichopithecus* aff. *arvernensis*. Together with the Vatera (Lesvos, Greece) and Karnezeika (Achaia) specimens^22,29^, it adds to the known *Paradolichopithecus* sample from Greece and can contribute to the broader discussion over the taxonomic affinities of the *Paradolichochopithecus*/*Procynocephalus* clade among Cercopithecidae^10–13,15,16,19^.

Here, we apply and critically discuss two distinct virtual reconstruction protocols on the facial complex of DFN3-150, with the main goal of better assessing its morphology and, subsequently, the degree to which the retrodeformation protocols may affect its systematic framework. In the initial step of this process we proceeded by virtually removing the sediment matrix from the cranium. In parallel, we assigned different bone fragments or individual parts of the cranium to dedicated segments^40^. Afterwards, we created two distinct manually reconstructed templates of the facial part of the cranium. To both these manually reconstructed templates and the original, two geometric morphometrics virtual reconstruction protocols were applied; (1) The Schlager and colleagues’ (2018) retrodeformation protocol^41^ and (2) the Amano and colleagues’ (2022) computerized restoration protocol^42^, with, and without surface semilandmarks.

In total, we produced nine individual virtually reconstructed models. Subsequently, a 3D geometric morphometric shape analysis allowed us placing all nine virtual reconstructions into a comparative framework restricted to macaques and *Papio* (as subtribal proxies), in order to investigate DFN3-150’s facial shapes’ morphometric affinities, in an allometric trajectories model perspective.

## Results

### Variation between reconstruction protocols

**Fig. 1:**
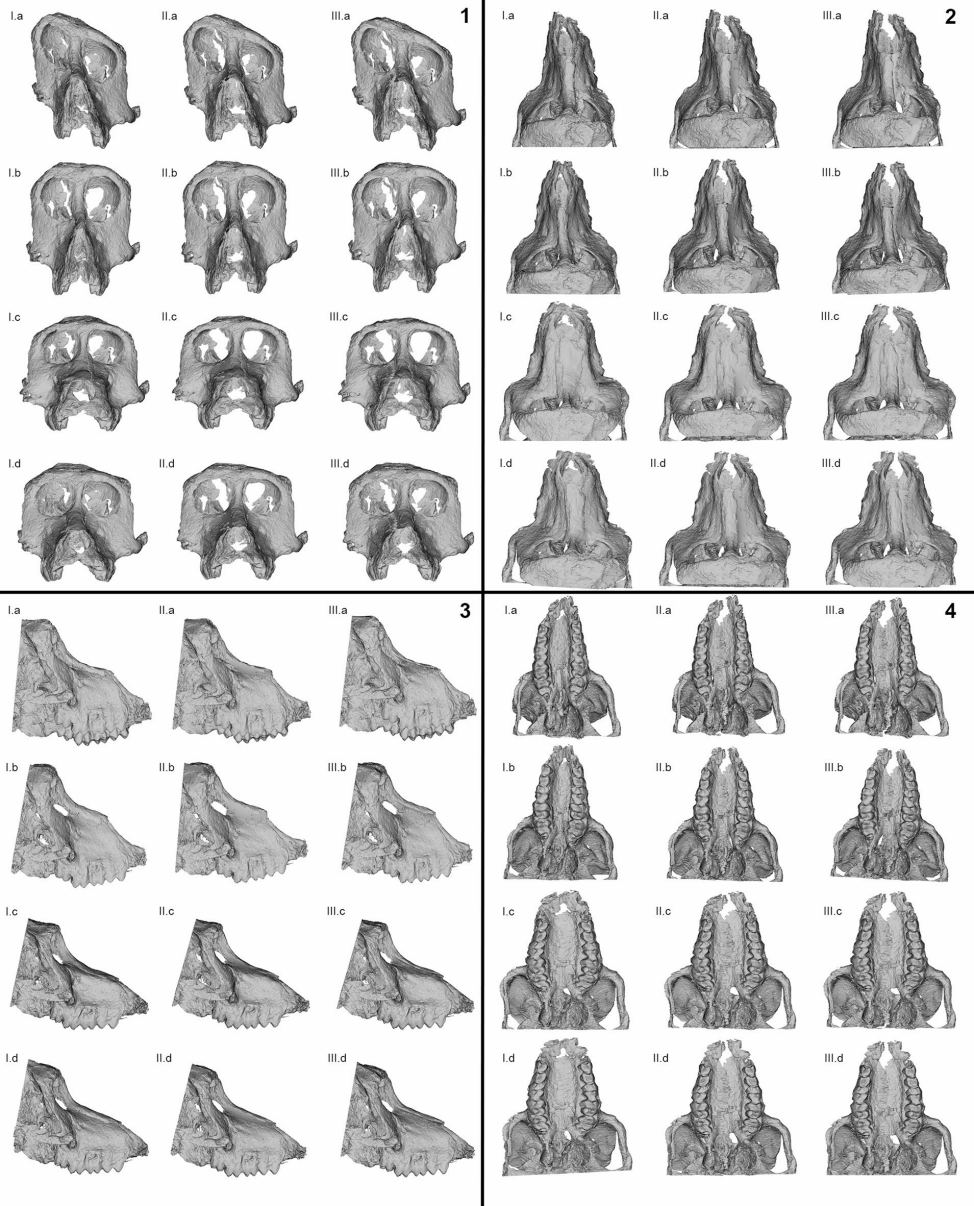
*Paradolichopithecus* aff. *arvernensis* DFN3-150 and subsequent reconstructions. **(I)** original fossil template; **(II)** ‘wideNasal’ reconstruction template; **(III)** ‘widePalate’ reconstruction template. **(a)** manual reconstructions; **(b)** retrodeformation after Schlager et al. (2018)^41^; **(c)** restoration after Amano et al. (2022)^42^ with surface semilandmarks; **(d)** restoration after Amano et al. (2022)^42^ without surface semilandmarks in (1) frontal, (2) dorsal, (3) right lateral and (4) ventral views quadrants. Differences between the virtual protocols mainly refer to a mediolateral compression of the models Schlager et al., 2018 protocol^41^, in relation those rendered via the utilizing the Amano et al., (2022) application^42^. This compression is evident in the shape of the orbits and the snout, as well as to the relative height and width of the models.

Applying different virtual reconstruction protocols to all templates (the original and the two manually reconstructed), produced substantially different forms (Fig. 1). Models following Amano and colleagues’ (2022) protocol^42^ display a wider rostrum, expressed ventrally and anteriorly, in both workflows (i.e., with and without surface semilandmarks). This protocol also affects orbital height, shortening the radius of the orbits. This issue arises from the application itself, since it depends on comparative specimens to restore a deformed target specimen. In contrast, Schlager and colleagues’ (2018)^41^ retrodeformations show the opposite effect. This may result from the initial deformation applied to the fossil.

The length of the rostrum remains relatively constant across the protocols, however, overall differences, such as its rectangular shape in the Amano and colleagues’ (2022) application^42^ with surface semilandmarks, highlight the impact of protocol choice. The lack of agreement among protocols in the shape of the orbits and nasal aperture further emphasizes this issue. Using multiple reconstruction strategies remains justified even for this specific fossil, where a strictly affine, unilateral deformation is not present.

### Principal component analysis (PCA)

**Fig. 2:**
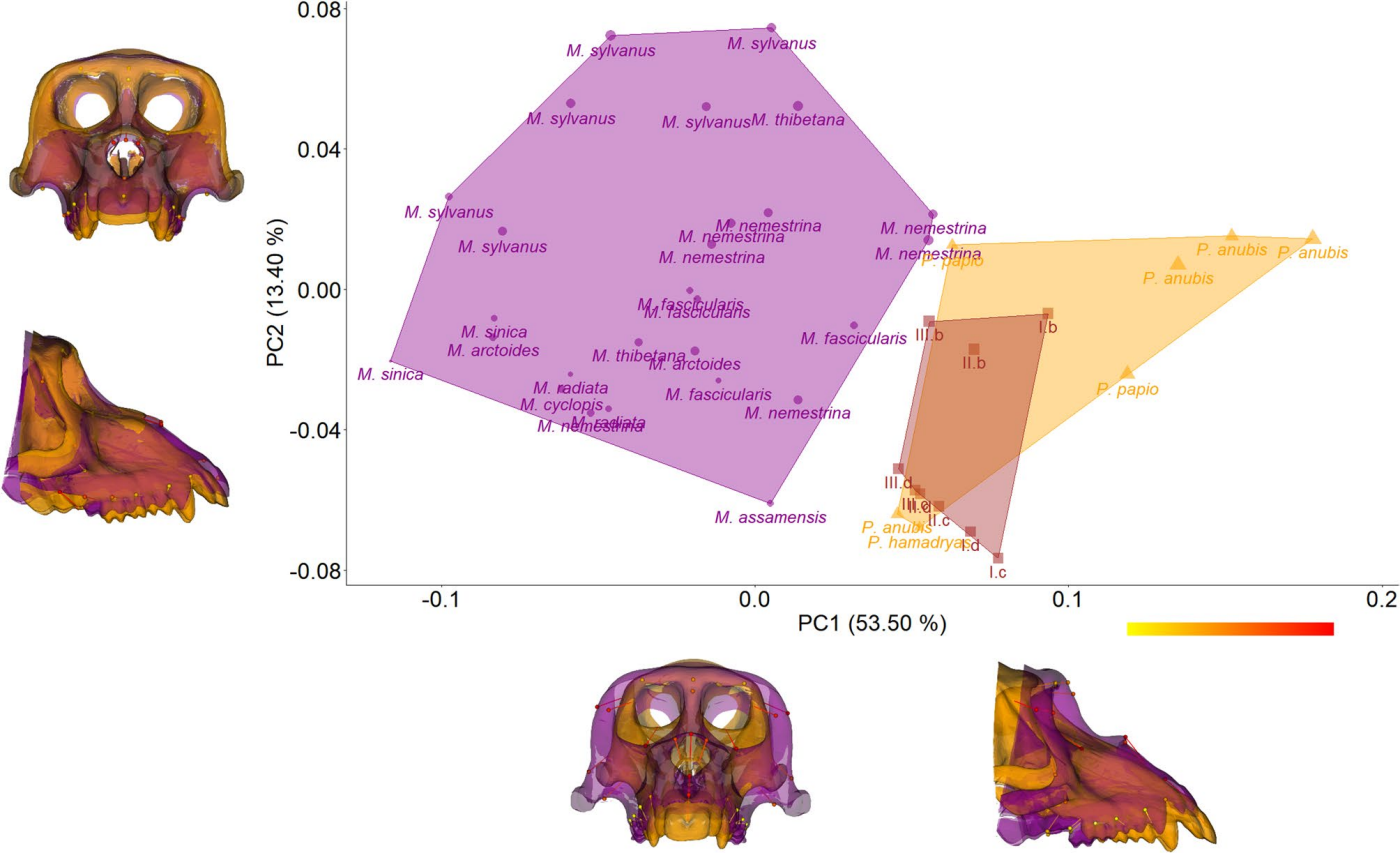
Principal component analysis (PCA) of the facial shape for papionine comparative samples and virtually reconstructed *Paradolichopithecus* aff. a*rvernensis*. The DFN3-150 *Paradolichopithecus* models are projected onto the PCA shape space. *Macaca* (*N* = 27) is shown in purple, *Papio* (*N* = 7) are shown in orange. *Paradolichopithecus* models are shown in brown. For a clearer visualization, abbreviations of the various DFN3-150 virtual reconstructions follow the Fig. 1 numeration; letters, b, c, d, correspond to the virtual reconstruction protocol and Roman numerals I, II, III correspond to the template upon which these protocols were applied. Symbol size reflects the relative centroid size of each specimen. Shape changes along PC1 and PC2 in frontal and right lateral views. Purple meshes depict the predicted shape in the minimum of each axis, and orange the maximum. Overlaid are the respective landmarks with the vectors of change. The color depicts the magnitude of variation of each landmark following the color scale (red maximum variation; yellow minimum variation).

The initial PCA plot in shape-space (Fig. 2) of the 33 anatomical landmark configurations (Supplementary Figure S6; Supplementary Table S2) revealed the influence of size on the overall ordination of specimens. The first three PCs summarized meaningful shape changes (threshold 1.62). Of these the first two PCs, which explain 66.9% of the total variance, separate the specimens into two major groups that reflect their generic division. PC1 primarily distinguishes macaques from *Papio*, the latter occupying the positive end of the axis. Specimens at the maximum of this axis exhibit a relatively long, downward-projecting rostrum, resulting in an overall narrower and taller cranium. This elongation reflects differential movements of specific landmarks, like rhinion, nasospinale, NPM and prosthion (Supplementary Table S2), that contribute significantly to the rostral and downward extension of the rostrum. In contrast, zygo-max superior, frontomalare orbitale and temporale direct elongation toward the posterior of the cranium with the vector pointing medially and anteriorly. At the positive extreme of PC1, the orbital margins appear relatively smaller but gain height at the root of the zygomatic, due to the medial displacement of these landmarks in parallel with the elongation vector.

On PC2, shape changes are not that pronounced and reflect variation within genera. From minimum to maximum values, landmarks with the highest variation are the NPM, rhinion, maximum zygomatic arch landmarks, all stretching the face posteriorly, but not as much as in PC1. Notably, the most variable landmark on this axis is distal M3. There, the vector of displacement points towards the inferior with a strong anterior component. This elongation vector, affecting the alveolar process, stops at the mesial P3 level. Beyond that, the rostrum recedes and slightly moves inferiorly (Fig. 2; Supplementary Figure S14). This particular condition is of a special weight in view of DFN3-150’s developmental age, for which the third molars are still erupting. An improved sample, increasing both late subadult as well as female individuals will be necessary to disentangle the implications of this observation.

Nonetheless, by showing positive scores on PC1 and negative on PC2, all nine DFN3-150 virtual reconstructions cluster nearer *Papio*. Only two *Macaca* specimens overlap with some DFN3-150 reconstructions on PC1 (cf. Figure 2). Along the PC2 axis, DFN3-150 templates reconstructed using the Schlager and colleagues’ (2018) protocol^41^ display greater variation compared to those reconstructed with the Amano and colleagues’ (2022)^42^, which are restricted to a more concentrated area on the lower values of PC2. Nonetheless, it is worth pointing out that all DFN3-150 models reconstructed according to Amano and colleagues’ (2022)^42^ protocol fall in the vicinity of the two late subadult *Papio* samples, one of which, *Papio anubis*, has been part of the reconstruction protocol.

### The effect of size variable

Even after the initial generalized Procrustes alignment (GPA), which removes isometric size effects^43^, the non-parametric two-tailed Kendall’s tau (τ) correlation test^44^ revealed a significant relationship between the first two PC scores and centroid size (PC1: τ = 0.55 and PC2: τ = 0.29 for p < = 0.01; Supplementary Table S12), while the test of homogeneity of multivariate dispersion revealed no significant differences among genera, with results above the acceptance threshold^45^ (*p* = 0.534) (Supplementary Table S13). This further allows us to interpret the allometric relationships between the two genera of the comparative material, whether they share a common allometric trajectory, or rather, each genus follows its own, unique shape to size relationship.

The unique allometries regression for the comparative samples, showed that the statistically not significant interaction term between size and genus, accounts for only 1.5% of the variation (Table 1; Rsq = 0.015, *p* = 0.43). In the subsequent common allometry regression, where the interaction was omitted, size explained 14.7% of the variation (Rsq = 0.147, *p* < 0.001), and genus explained 10% after accounting for size (Rsq = 0.1, *p* < 0.001) (Table 1). Both main effects were highly significant. The vector correlation test that we used to evaluate the homogeneity of angles between slopes, further supports the common allometric model (Z-score = 0.55, *p* = 0.29; Supplementary Table S15), indicating approximate parallel slopes. This is particularly important, since the overlap among predictors (i.e., size and genus) is not extensive. The variance inflation factor (VIF) is 1.86, and the tolerance 0.55, indicating a moderate linear relationship between size and genus, verifying their distinct predictive reliability. This applies even in the presence of a high correlation of size to genus. However, it has to be noted that sex, age and species status of the specimens were not explicitly modeled, and thus may further contribute to residual variation.

**Table 1:** The permutational MANCOVA results of the multivariate regression of tangent space coordinates of the comparative sample for the unique and common allometries models respectively.

		Df	SS	MS	Rsq	F	Z	Pr(> F)
Unique	Size	1	0.046	0.046	0.147	8.91	3.655	< 0.001
	Genus	1	0.034	0.034	0.109	6.647	3.515	< 0.001
	Size: Genus	1	0.004	0.004	0.015	0.933	0.16	0.43
	Residuals	30	0.155	0.005	0.495			
	Total	33	0.313					
Common	Size	1	0.046	0.046	0.147	8.92	3.656	< 0.001
	Genus	1	0.034	0.034	0.10	6.661	3.520	< 0.001
	Residuals	31	0.160	0.005	0.51			
	Total	33	0.313					

### The effect of grouping variable

Whether shared allometry reflects a true biological pattern or is potentially influenced by the smaller *Papio* sample (*N* = 7), the rarefaction analysis (Supplementary Table S16) resulted in a median p-value of 0.43. Out of 1,000 replicates, 38 (3.8%) resulted in an interaction with a p-value below the α-level of 0.05. This indicates that even with balanced sample sizes, there is no significant genus-by-size interaction. In contrast, null permutations of genus labels within each rarefied set, resulted in a higher median p-value (*p* = 0.82). Out of 20,000 null permutations, 134 iterations (0.67%) resulted in a p-value below the α-level threshold for the null hypothesis of parallelism. Across all iterations, Z-scores of the interaction term retain low and positive values, while null distribution Z-scores are low and negative (Supplementary Figure S15). This indicates a coherent structure of the data, which disappears completely when group assignments are randomized.

Nevertheless, these results differ from those obtained with phylogenetic MANCOVA (Supplementary Table S17), where only the size predictor had a significant effect on facial shape (*p* < 0.001), while genus was not significant after accounting for shared evolutionary history (*p* = 0.99). Given the interaction between these variables was initially significant in the phylogenetic model (*p* = 0.018), the GLS rarefaction analysis demonstrated that this effect was not robust to differences in sample size between genera; when group sizes were balanced, the interaction term became non-significant (median *p* = 0.88; Supplementary Table S18). Only 15 out of 1,000 replicates (1.5%) resulted in a p-value below the threshold. Across the null permutations, 7197 out of 20,000 (35.98%) tests yielded a p-value below threshold, indicating that slope deviations are more likely when the genus predictor is shuffled than in the observed rarefied data. This is reflected in the Z-score distributions (Supplementary Figure S15) and suggests that genus-level differences in facial shape are largely the result of phylogenetic divergence among taxa. Consequently, genus is verified as a predictor that reflects evolutionary relationships in facial shape, and a hypothesis of common allometric trajectories is suggested.

### Common allometry analysis: size and genus effect differences

**Fig. 3:**
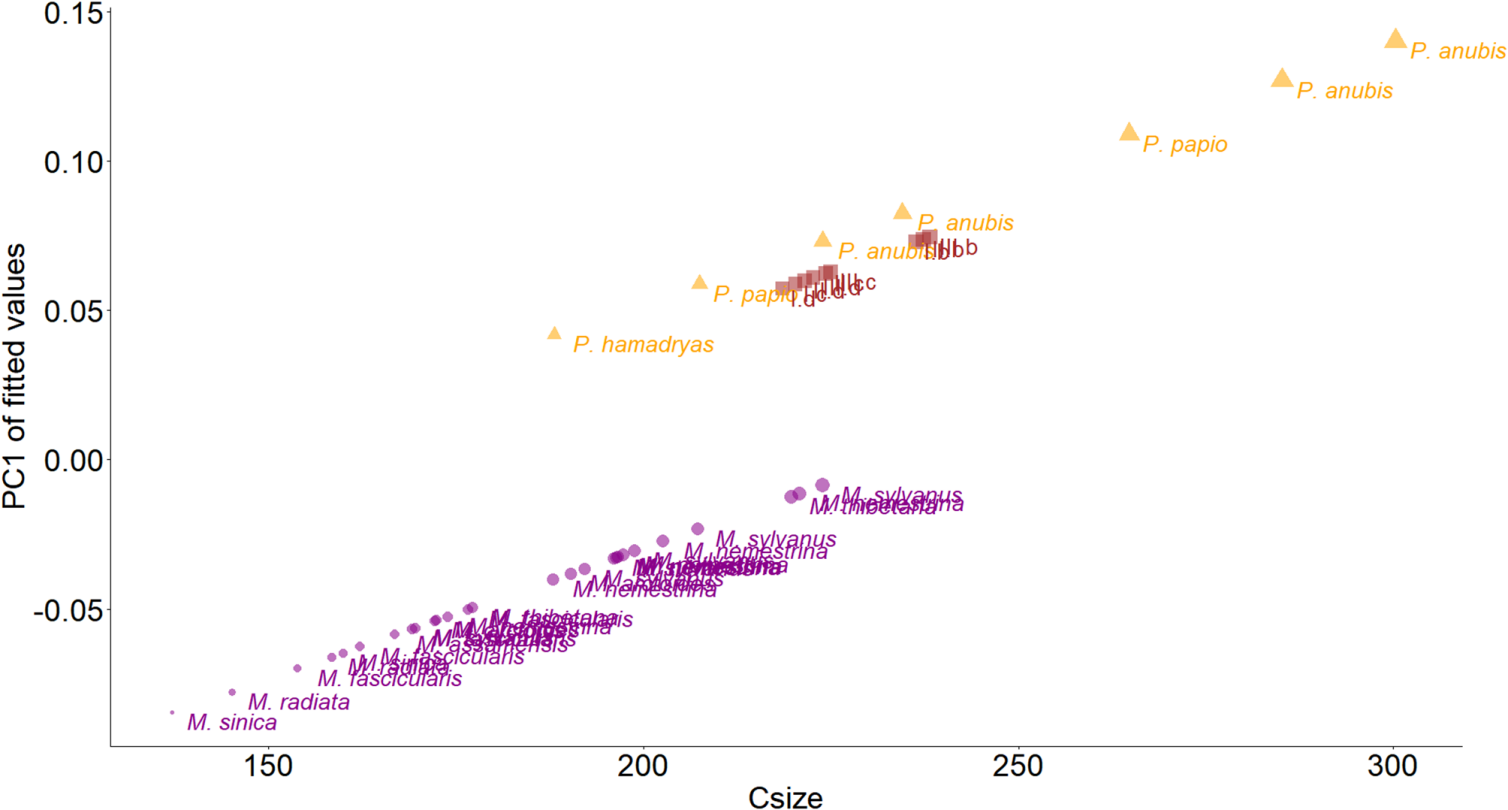
PC1 of fitted values of the common allometry regression plotted against centroid size. In purple are *Macaca*, orange are *Papio*, and in brown are *Paradolichopithecus* models. Centroid size differences are graphically displayed with the symbol’s size. For a clearer visualization, abbreviations of the various DFN3-150 virtual reconstructions follow the Fig. 1 numeration; letters b, c, d, correspond to the virtual reconstruction protocol and Roman numerals I, II, III correspond to the template upon which these protocols were applied.

Alongside the variation induced by centroid size, genus-specific differences must not be ignored (Rsq = 0.1, Z = 3.52, *p* < 0.001). This suggests that evolutionary shape variation is linked to overall phylogeny. Nevertheless, as stated above, no other potentially contributing factor has been explicitly modeled, and therefore, these results should be considered as preliminary. The PC1 scores of the fitted values -predicted shape component of the common allometry regression (no interaction between terms) - (Fig. 3), accounting for 74% of the variation, when plotted against centroid size show significant and consistent proximity of *Paradolichopithecus* reconstructions to *Papio* (shorter intercept difference). The opposite motive appears in a definite way when we plot the PC2 of fitted values against centroid size, which accounts for 20.3% of the variation (Supplementary Figure S16), suggesting that this axis, as predicted by size and genus, captures shape differences attributable to size effect alone. This conclusion is primarily deduct by the fact that genera overlap on PC2 of fitted values axis, while the overall effect of size on shape is still apparent.

**Fig. 4:**
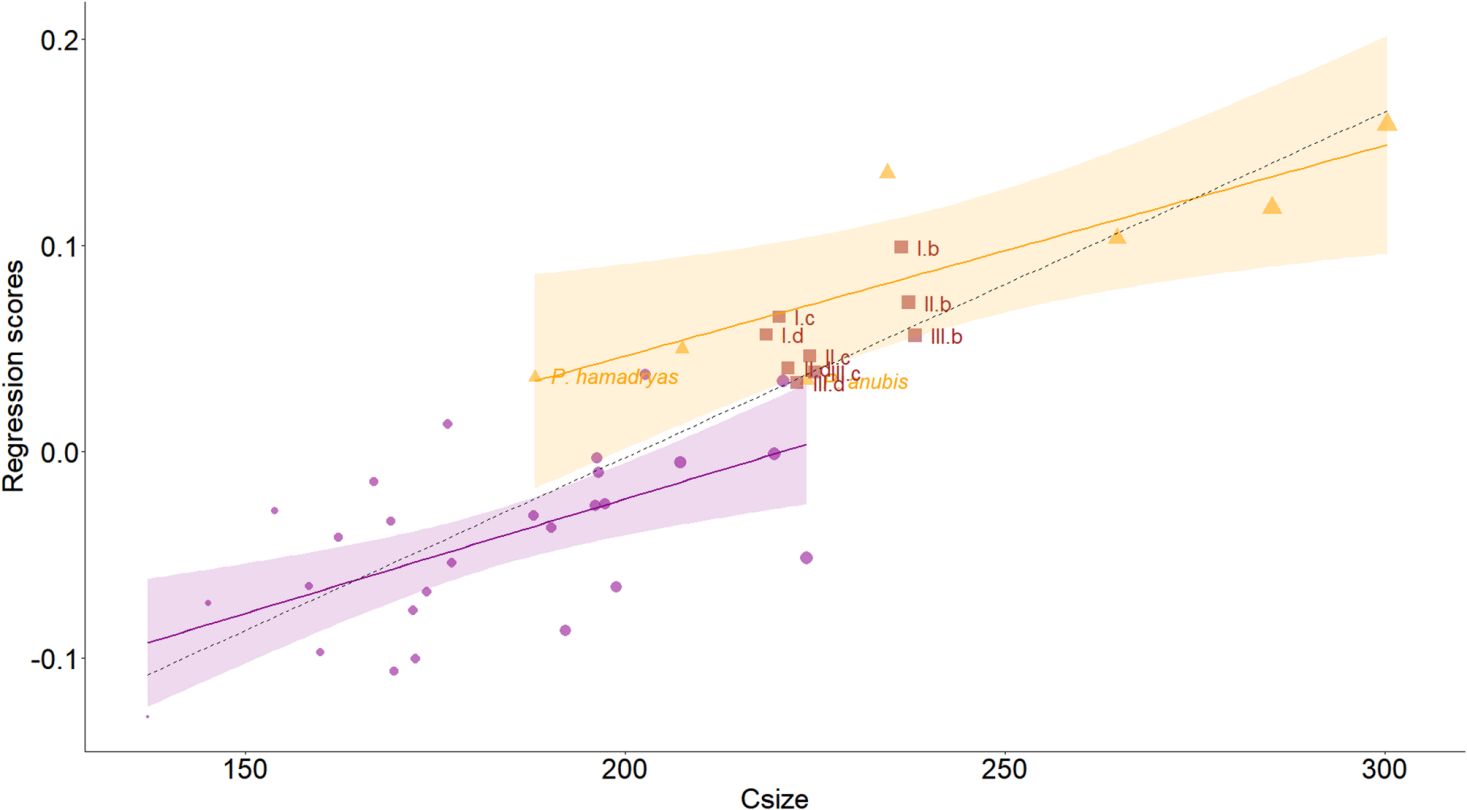
Regression scores of the regression with centroid size as the sole predictor (simple allometry), plotted against centroid size. In purple are *Macaca* species, in orange *Papio*, and in brown *Paradolichopithecus* models. The purple and orange solid lines are the *Macaca* and *Papio* individual regression lines respectively. Around the lines is the 95% confidence interval area. The black dashed line is the shared allometry regression vector. Centroid size differences are graphically displayed with the symbol’s size. For a clearer visualization, abbreviations of the various DFN3-150 virtual reconstructions follow Fig. 1 numeration; letters, b, c, d, correspond to the virtual reconstruction protocol and Roman numerals I, II, III correspond to the template upon which these protocols were applied. Abbreviations in the rest of the comparative material has been omitted. Subadult *Papio* specimens are indicated by their abbreviations.

The same conclusion is held when examining shape discrepancies alongside their static allometric component, plotted against centroid size (Fig. 4). *Macaca* species mainly occupy the negative values of the regression scores, while *Papio* and *Paradolichopithecus* the positive. Alternatively, the degree of centroid size overlaps among genera, ranging from moderately small to larger adult *Macaca* specimens and smaller *Papio*, such as subadults or females (or both), supports the argument of inherited shape differences. *Paradolichopithecus* models rendered with the Amano and colleagues’ (2022) protocol^42^ fall within the range of the largest macaques and the smallest to medium-sized *Papio*, with a slight overlap in regression scores, while the ones created with the Schlager and colleagues’ (2018) protocol^41^ do not display an exceptionally different pattern. Again, however, models rendered by Amano and colleagues’ (2022) protocol^42^, are closer to the *Papio anubis* specimen used to restore DFN3-150. Beyond that, a denser sampling in the range of overlap, in relation to the sex and age of DFN3-150 is advisable for future studies, and could possibly clarify potential flaws, recalibrating the overall allometric trajectory.

As regression scores move towards positive values, shape changes refer to the general elongation of the rostrum and heightening of the face, as is well documented^9,30,31^. The elongation is differentially driven via a downward projection of rhinion, prosthion, and nasospinale, followed by a lesser downwards movement of the alveolar process landmarks, as far as the M1-M2 contact. At the same time, landmarks sampled on the zygomatics and frontal bone are subtracted from the face with a relative medial direction for the bilateral ones, giving the narrow appearance to the face (Supplementary Figure S17).

However, when we further partition variation and plot shape scores of the common allometry model, complications become more pronounced (Supplementary Figure S18). Shape similarity among mid-range centroid sizes is strong, with some *Papio* individuals and all *Paradolichopithecus* reconstructions scoring alongside medium to large-sized macaques. This is the additive effect of genus. Here, both *Papio anubis*, along with DFN3-150, are categorized as female subadult individuals^10^, *Papio hamadryas* specimen refers to a subadult of unknown sex and one of two *Papio papio* is an adult female individual. As it becomes apparent, age as a factor does not obscure the overall trend, but at the same time the genus effect is highlighted. Nevertheless, conditioning on genus, an evolutionary perspective emerges, as the genera are discriminated on their common allometric vector.

### Mean shape differences associated to genus

After accounting for common allometry, the predicted mean shapes of each genus at the fixed comparative sample mean centroid size exhibit clear separation (Supplementary Figure S19). Holding the size covariate constant in these predictions ensures that the only source of mean shape difference among groups is the genus effect. Principal component analysis of these predicted genus least squares means revealed that *Macaca*, *Papio*, and *Paradolichopithecus* reconstructions, each occupy distinct regions of shape space on the first two principal components. *Papio* and *Paradolichopithecus* lay on the positive values of PC1, which describes most of the variation (PC1: 76.82%, PC2: 23.18%). The 95% confidence ellipses around each predicted mean demonstrate no overlap, thus indicating robust genus-level shape differences independent of size. These results confirm that a shape component is predictable through genus-level differences.

Unlike the pronounced facial elongation in size-related changes, genus-level differences are characterized by shifts in the relative positions of specific landmarks. Towards the direction of maximum PC1, rhinion, NPM, and prosthion, collectively displace the anterior-most portion of the rostrum downwards, while other facial landmarks — such as zygo-max superior, zygo-max inferior, and the point of maximum zygomatic arch — move ventrally, effectively contracting the midface. In contrast to allometric trends, the mesial and distal M3, as well as M1-M2 landmarks all shift caudally, though to different extents, and distal M3 slightly ventrally; notably, distal M3 exhibits a disproportionately longer displacement vector further contributing to a relatively more pronounced tuber maxillae. While these results are important on their own, the degree to which the terminal position of the M3 for all subadult individuals (i.e., all DFN3-150 reconstructions and two *Papio* samples) would alter them remains unknown and a more developmentally diverse sample is required to further explore ontogenetic effects. Nonetheless, all *Macaca* and most *Papio* specimen are represented by adult individuals, thus, the current polarity maintains a certain degree of validity. On the other hand, landmarks on the cranial vault primarily shift rostrally, with a marked elevation component, but exhibit relatively less variation overall (Supplementary Figure S20).

## Discussion

Here, we present multiple reconstructed models of the DFN3-150 cranium to allow for the evaluation of its morphological affinities and to explore the effect of the reconstruction protocol on its perceived affinities. The reconstructed models are based on three manually reconstructed templates and two distinct virtual protocols^41,42^. Although the reconstructions have not been experimentally assessed for their validity, they all, to varying degrees, correct for plastic deformations (cf. Figure 1).

Qualitatively assessing the reconstruction protocols’ performance, the algorithm provided by Schlager and colleagues (2018)^41^ should not be considered as producing a trustworthy representation of the antemortem form of DFN3-150. We applied this protocol as a taxonomically unbiased method to assess bilateral deformation. However, due to its specifications, it cannot be considered adequate for specimens with unilateral deformation^41^ signal. This misuse in the current study is more apparent in the shape space PCA (Fig. 2). All three templates, retrodeformed using this approach, appear inconsistent with the models restored by the other protocol^42^, both in shape space and in the regressions. They likely retain some degree of deformation signal from the manually reconstructed templates. This is particularly evident in the orbital part of the face, while the rostrum seems less affected.

Conversely, the protocol suggested by Amano and colleagues (2022)^42^ introduces a significant amount of variation from the reference specimens, making its application debatable, especially when considering the broader *Paradolichopithecus* discussion^10,16,23^. Thus, the most conservative models are those generated using the Amano and colleagues (2022) protocol^42^, where surface semilandmarks were not considered. This aligns with the general view of other adult *Paradolichopithecus* crania, both males and females, where the maxilla outline at a coronal plane forms an ellipsoid^13,46,47^.

This peramorphic structure stands after considering sex differences and age, as ontogenetic shape changes between sexes are not significant before adulthood^32^. This holds true despite the noticeable increase in the size of *Paradolichopithecus* crania in an evolutionary context^14^. The latter approach led to a precise and controlled restoration of the upper viscerocranium, focusing on the morphological aspects of the orbits while preserving much of the original shape of the rostrum.

Another shortcoming not explicitly addressed in the current study concerns the developmental age group of DFN3-150. Using adult specimens in an evolutionary context allows for the interpretation of the full phenotypic expression^33^. To overcome this limitation in future reconstructions, we propose implementing an ontogenetic vector of allometry from extant relative species, potentially including additional phylogenetic information^48^, while also incorporating available adult forms of *Paradolichopithecus*.

By obtaining this, it could be possible to simulate the adult form of young individuals and inversely. At the same time, one could reconstruct DFN3-150 using tighter phylogenetic and geochronological constraints in a “forward ontogenetic projection”^49^. This approach could also focus on specific areas of the cranium, reflecting regional changes that correspond to the reference sample. This way we could avoid a broad extrapolation of species’ differential ontogenetic paths in the peramorphic form of the target. A “lesser scaled” regional approach may also be sufficient to distinguish the phylogenetic signal^50^ surpassing homoplastic effects.

To test the effect of these described differences in the reconstructed models of DFN3-150 onto its perceived morphologic affinities, we explored overall morphological similarities in a comparative framework of *Macaca* and *Papio*. While the *Paradolichopithecus*-Macacina sister clade relationship remains the leading hypothesis in *Paradolichopithecus* systematics^10,14,16^ (and references therein), the exclusion of baboons from the discussion has not yet been guaranteed^15,24,27^. To explore this, we preliminary examine the established hypothesis that the *Paradolichopithecus* clade evolved in situ from an ancestral European *Macaca* population^14^ by showing a trend of increasing size.

Given the high correlation between centroid size and body mass, at least for Papionina^7,30,31,33,51^, allometric scaling plays a significant role in shape variation in papionins. On that behalf, size broadly reflects evolutionary relationships among the currently provided comparative species. Given the evolutionary importance of centroid size and the allometric relationships affecting cranial form of Papionini^30,31^, our data, when shape variation is modeled as a strict function of size and genus, shows that the DFN3-150 reconstructions cluster in two groups (Fig. 3); the reconstructions following the Schlager and colleagues’ (2018) protocol^41^ (greater centroid sizes) and the reconstructed models resulting from the Amano and colleagues’ (2022) protocol^42^ (smaller centroid sizes). Nevertheless, DFN3-150 models plot along the allometric trajectory of our *Papio* sample. This suggests that DFN3-150 reconstructed models are morphologically closer to *Papio* than to macaques, beyond what would be the result of allometry.

This hypothesis is not a direct evolutionary inference, but rather a hypothesis that should further be examined with a more inclusive comparative sample (in terms of ontogenetic age, sex, genus and species, especially of Papionina). To further enable comparability between studies with regard to the influence of allometry on taxonomic affinities within Papionina, a combined dataset of morphometric and non-metric data would be required. Moreover, ontogenetic allometric effects for *Papio*^33^, and sexual dimorphism in the overall comparative sample were not considered in our study. It is also important to note that only two comparative specimens represent late subadult individuals, which are already close to their expected adult morphology. Similarly, DFN3-150^10^ has also been described as a subadult individual. While levels of sexual dimorphism in juvenile individuals are not expected to substantially differ, we deliberately did not pool the genera for sex, so as to maintain as much variation as possible. We based our choice on the fact that intraspecies variation attributed to sex is a product of overlapping ontogenetic allometries^52–54^, and differences between sexes manifest as additional secondary trajectory deviations^53^ at later ontogenetic stages. Thus, at a tribal level, apparent differences within genera are not expected to influence evolutionary allometric inferences^54^. Beyond that, the extension of male size ranges in *Papio* is expected to further contribute to phenotypic variability in shape space, thus allowing for an appropriate overall allometric trajectory calibration. This is especially important in a case of a rather restricted comparative sample, as is the current *Papio* one.

The proximity of DFN3-150 reconstructions near *Papio* in the modeled multifactor regression context, or at least their intercept distance from *Macaca*, suggests that it shares closer morphological affinities with *Papio* at any given size. This is particularly relevant when considering the shared ontogenetic trajectory of both genera^30^. The latter is reflected in Fig. 4, where our restricted subadult *Papio* sample approximates the shape of adult macaques. Centroid sizes of the largest macaques, including the conservative^55,56^ *Macaca sylvanus*, which represents the ancestral state of this subtribe, and smaller Papionina overlap. Thereby, it can be assumed that DFN3-150 may have already reached score ranges beyond adult *Macaca*.

This is especially true when we consider that larger sizes are expected to lead to new shape spaces in the Papionini^30,31^, a process also influenced by biological age^51^ in female *Papio*. Thus, the still erupting M3s in subadult *Papio* specimens and possibly DFN3-150, are expected to further alter facial shape. This change coincides with the corresponding allometric trajectory but maintains the M3 position relative to the temporomandibular joint^57^. Regardless, ontogenetic allometry may not be indicative of homology, and an ontogenetic dissociation among Papionini has been previously documented^54^. In the present study, we identified the distal M3 as the most variable landmark between the two examined genera, portraying a different direction, as opposed to the static allometric effect on it (Supplementary Figure S20).

For this study, we deliberately excluded other effects, such as sex, age, and broader ecological and functional aspects of residual phenotypic expression^31,33,51^, to avoid an extended collapse of the allometric signal. This can be seen in the common allometry regression shape scores (Supplementary Figure S18), where individuals appear to become more mixed over a certain range as variation is further partitioned, highlighting the offset shape differences among genera. This restriction, though, is of primary concern and makes any systematic evaluation of the results preliminary in the broader evolutionary perspective.

The main areas affected by allometry, as centroid size increases, concern a relative contraction of the maxilla and the frontal process of the zygomatic. This extends to the root of the zygomatic process of the frontal bone, with a milder depression towards smoother anteorbital drops in larger specimens. Shape signals not linked to these changes likely result from other factors, possibly reflecting broader phylogenetic relationships *sensu lato*. In any case, the allometry controlled prediction of mean shape differences among all genera, highlights the proximity of DFN3-150 to *Papio* spp., and by extension possibly Papionina; a systematics alternative ought to be further evaluated.

## Conclusion

The here-presented reconstructed models of DFN3-150 exhibit distinct morphological differences due to differences in the underlying reconstruction protocols. This is most pronounced with regard to centroid size, which is systematically larger in reconstructions according to the Schlager and colleagues’ (2018) protocol^41^, and orbital and rostrum shape (cf. Figures 1 and 3, and 4). Although our analyses yielded consistent results across all reconstructed models, reconstructions following the Amano and colleagues’ (2022) protocol^42^ should be considered the most plausible and provide a solid base for future in-depth analysis of DFN3-150’s taxonomic affinities.

## Materials and methods

### Materials and comparative sample

In addition to the fossil of interest DFN3-150, our sample included 34 non-pathological specimens: 27 members of the Macacina subtribe and seven members of *Papio* (Papionina) (Table 2; see Supplementary Table S1 for detailed sample information). The single *Mandrillus sphinx* specimen listed in Table 1 does not contribute to the comparative sample for statistical analysis, but was part of the virtual reconstruction protocol^42^.

**Table 2:** Specimens used in this study as comparative material. *Mandrillus sphinx is used in the virtual reconstruction protocol^42^ only.

Species	Catalog Number	Repository	Sex	Dental score	Abbreviation
*Macaca arctoides*	USNM 111966	NMNH	Female	5	*M. arctoides*
*Macaca arctoides*	USNM 256825	NMNH	Male	5	*M. arctoides*
*Macaca assamensis*	USNM 15255	NMNH	Female	5	*M. assamensis*
*Macaca cyclopis*	USNM 296795	NMNH	Male	5	*M. cyclopis*
*Macaca fascicularis*	USNM 573504	NMNH	Male	5	*M. fascicularis*
*Macaca fascicularis*	USNM 317191	NMNH	Male	5	*M. fascicularis*
*Macaca fascicularis*	USNM 121511	NMNH	Male	5	*M. fascicularis*
*Macaca fascicularis*	USNM 256072	NMNH	Female	5	*M. fascicularis*
*Macaca nemestrina*	USNM 241022	NMNH	Male	5	*M. fascicularis*
*Macaca nemestrina*	USNM 114502	NMNH	Female	5	*M. nemestrina*
*Macaca nemestrina*	USNM 123144	NMNH	Male	5	*M. nemestrina*
*Macaca nemestrina*	USNM 154367	NMNH	Male	5	*M. nemestrina*
*Macaca nemestrina*	USNM 399506	NMNH	Female?	5	*M. nemestrina*
*Macaca nemestrina x mulata*	USNM 396929	NMNH	Female	5	*M. nemestrina*
*Macaca radiata*	USNM 398463	NMNH	Female	5	*M. radiata*
*Macaca radiata*	M-163078	AMNH	Male	5	*M. radiata*
*Macaca siberu*	USNM 546835	NMNH	Male	5	*M. siberu*
*Macaca sinica*	USNM 15259	NMNH	Male	5	*M. sinica*
*Macaca sinica*	USNM 271190	NMNH	Female	5	*M. sinica*
*Macaca sylvanus*	USNM 255979	NMNH	Male	5	*M. sylvanus*
*Macaca sylvanus*	USNM 476782	NMNH	Female	5	*M. sylvanus*
*Macaca sylvanus*	1392	Japan Monkey Center	Male	5	*M. sylvanus*
*Macaca sylvanus*	6330	Japan Monkey Center	Male	5	*M. sylvanus*
*Macaca sylvanus*	I	iPHEP, Uni. of Poitiers	Male	5	*M. sylvanus*
*Macaca sylvanus*	USNM 476780	NMNH	Male	5	*M. sylvanus*
*Macaca thibetana*	USNM 241162	NMNH	Female	5	*M. thibetana*
*Macaca thibetana*	USNM 241163	NMNH	Male	5	*M. thibetana*
*Mandrillus sphinx*	3091	PRI, Kyoto Uni.	Female	4->5	*-*
*Papio anubis*	3086	PRI, Kyoto Uni.	Female	4	*P. anubis*
*Papio anubis*	1626	PRI, Kyoto Uni.	Male	5	*P. anubis*
*Papio anubis*	USNM 162899	NMNH	Male	5	*P. anubis*
*Papio anubis*	USNM 397476	NMNH	Female	5	*P. anubis*
*Papio hamadryas*	USNM A 49732	NMNH	?	4	*P. hamadryas*
*Papio papio*	USNM 378669	NMNH	Male	5	*P. papio*
*Papio papio*	USNM 381430	NMNH	Female	5	*P. papio*
***Paradolichopithecus*** **aff.** ***arvernensis***	**DFN3-150**	**LGPUT**	**Female**	**4**	*-*

The comparative sample is predominantly comprised of adults but not elderly (dental score 5) specimens with the exception of three subadults, of which two show dental score^39^ 4 like DNF3-150 and one the transition to dental score 5. Further, to account for sexual dimorphism, the comparative sample includes specimens of both sexes (13 female, 20 male), one of unknown sex and one possibly female.

Virtual surface models for the majority of the comparative sample were retrieved from the Smithsonian Institute digital primates collection (www.vertebrates.si.edu, Washington), the Primate Research Institute, Kyoto University (www.dmm.pri.kyoto-u.ac.jp, KUPRI, Kyoto), and the Morphosource online database (www.morphosource.org). Additionally, one adult male *Macaca sylvanus*, scanned in the same facilities as DFN3-150^10^, was made available for this study by PALEVOPRIM (Université de Poitiers, France). No animals reported here were harmed or killed for the purpose of this study.

### Segmentation and reconstruction protocol of DFN3-150

#### ***µ***CT scan and virtual segmentation

The DFN3-150 *Paradolichopithecus* aff. *arvernensis* cranium underwent a micro-computed tomography (µCT) scan at Plateform Platina (IC2MP, Université de Poitiers) in Poitier, France. The resulting acquisition comprises 1771 slices, each with an isometric voxel size of 0.07 mm^10^. microCT technology allows for the conversion of material with varying coefficients of absorption into corresponding grayscale variations in an image. This image, in turn, is referred to as a tomographic slice and allows for a virtual representation of the scanned object with the use of dedicated software.

The initial inspection of the CT slices revealed three major patterns of grey value ranges concerning the bony tissue and matrix transition (Supplementary Figure S1, S2). This condition constitutes a source of error due to subjective manual segmentation is required in areas where no (semi-) automated threshold-based segmentation is possible. Grey value differences between materials are substantially reduced in the posterior part of the neurocranium. Segmentation in this area is further complicated by the collapse of the inner table in the parieto-occipital area around the nuchal crest. Due to the collapse, bone fragments lie inside the matrix infilling the braincase and show very little difference to the surrounding matrix. Similarly, the viscerocranium, especially in the frontonasal region, shows a complex, overlapping pattern of grey values between bone and surrounding matrix materials.

For the initial segmentation step^58^, the main working plane we used is the sagittal one, and the corrections were made in the coronal and transverse planes. Segmentation design is meant to maximize the number of extracted segments, to increase the number of segments that can be moved independently throughout the subsequent manual reconstruction. While only a relatively small portion of the cranium appearing fragmented in the neurocranium^10^, most of the observed taphonomic disturbance is related to plastic deformation. In order to eliminate as much of this deformation as possible, already during the first steps of reconstruction, segmentation was guided both along breakage patterns as well as sutures, or, otherwise, areas treated as suture zones. In addition, the frontal bone was separated into two segments to account for a compression.

A strong compression in the right supraorbital torus around the zygomatic process can be observed, which is accompanied by a relatively thickened diploe. Another more difficult to interpret inner structure that superficially appears like a “boundary line” emerges in the same area and divides the rostral part of the frontal bone into three parts: two – right and left – “superciliary” areas and a central “ophryonic” in between them (Supplementary Figure S3). A metopic suture (as expected for the specimens’ dental group) or other fracture is absent; thus, this structure is likely the outcome of compression rather than any anatomic trait. The division of the frontal bone into two segments was applied on this latter rightmost compression zone.

**Fig. 5:**
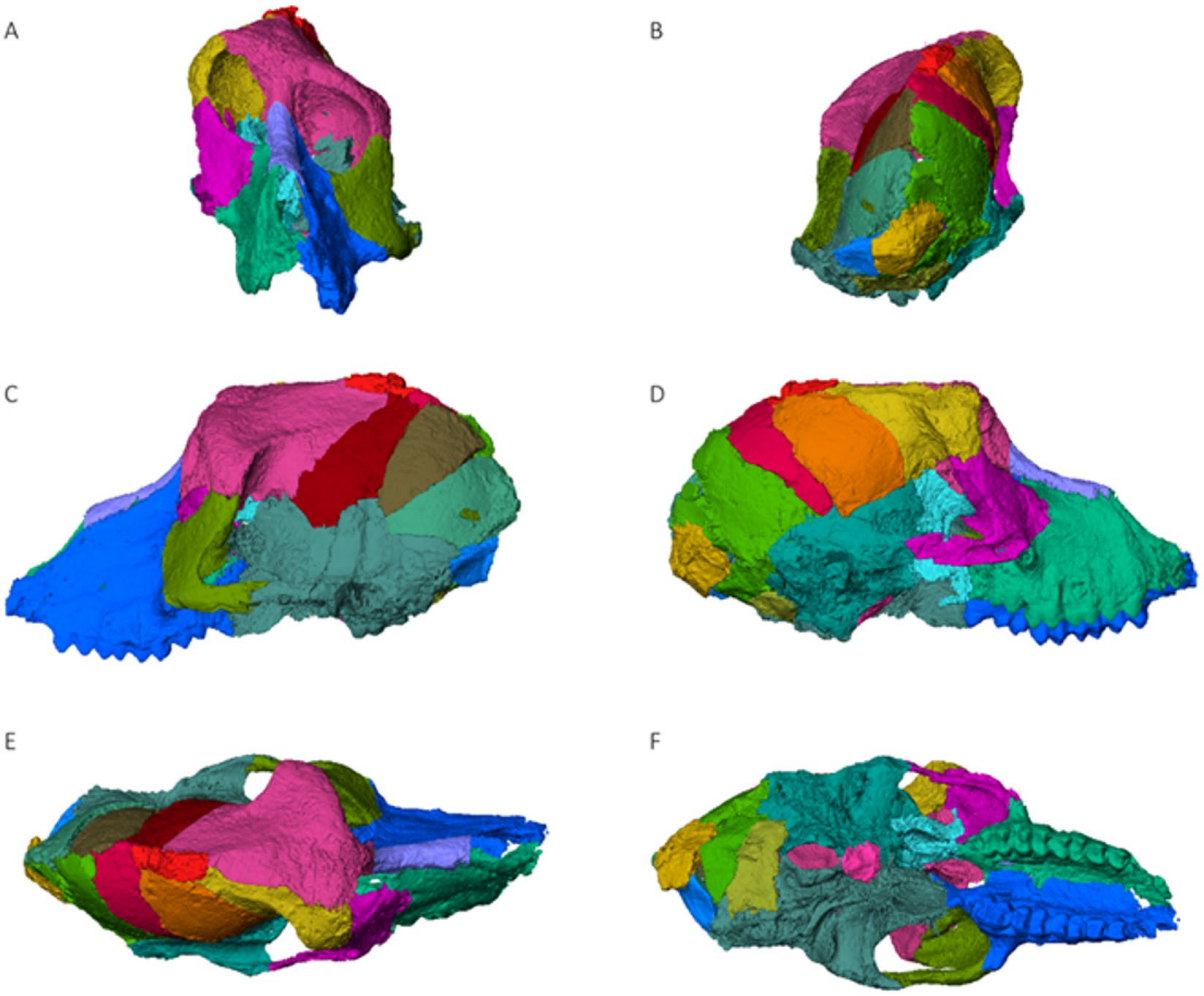
The segmented cranium. ***A***) frontal; ***B***) caudal; ***C***) left lateral; ***D***) right lateral; ***E***) dorsal; ***F***) ventral views. Unscaled.

The initial segmentation of the full stack of slices yielded 36 virtual segments, of which 25 were retained following the selection process (Fig. 5). We integrated the remaining 11 small fragments into adjacent larger segments or completely omitted them due to difficulty in their manipulation. The viscerocranium comprises five segments, while the neurocranium comprises 20 segments, one of which individually accounts for the sella turcica. Some fragments do not share bilateral counterparts, i.e., the nasal bone fragment and the protruding calvaria top, which were neither assigned to the frontal nor parietal segments, respectively.

### Manual reconstruction

The original specimen appears compressed dorso-laterally, with its right bilateral side being mainly affected. This lateral compression is mostly evident in two areas. First, the overlay of the right maxillary palatine process (for which no transverse palatine suture is evident and so is merged with palatine bone) on its left bilateral counterpart, generally following the median palatine suture orientation (Fig. 5; Supplementary Figure S4), and second, the right maxilla, where the entire frontal process of the maxilla overlays on the nasal fragment (Fig. 5; Supplementary Figure S4). This compression results in the vertical elevation of the right hemicranium as opposed to its left counterpart. We base the validity of these reconstructions on the segments’ smoothness criterion^40^.

*Manual reconstructions.* We manually corrected these described deformations individually by producing two separate models; the “wideNasal” model for the correction of the nasal overlay over the right maxilla (Fig. 1 IIa), and the “widePalate” version for correcting the right-to-left palatal overlay (Fig. 1 IIIa; for an overview of the whole pipeline followed in this study see Supplementary file SOM2). We refer to these models as “decompression templates” as they appear relatively widened compared to the original specimen. These models, which resulted in the lateral decompression of the cranium, will, in addition to the original fossil, serve as templates for subsequent digital reconstruction protocols.

For the “widePalate” template, we manually translated and rotated the right maxillary segment to align it with its left counterpart. This process was controlled by the estimated width of the distal part of the horizontal plate of *os palatinum*. The two opposite parts of this edge should be bisected by the staphylion (Supplementary Table S2) landmark, which is the midpoint located on the tangent to the posterior notches of the horizontal plate of *os palatinum*. Transformation stopped when the line marking the posterior end of the palate became perpendicular to the palatine suture, passing through the staphylion landmark. Then, the translation and rotation values of the right maxillary segment were retrieved and applied to all other segments from the right cranial side, ensuring they smoothly followed the overall transformation, assuming that there is only one field of homogeneous vectors that impact similarly the right-side segments.

We applied the same approach for the “wideNasal” reconstruction. In this case, the goal was to move the right maxilla segment to its correct anatomical position. We focused on recreating the continuous contour along its frontal process, prioritizing the smoothness of the transition over the exact extent of the fusion between the two fragments. We then applied the same translation and rotation values for the maxillary segment to the other two viscerocranium segments and all other fragments from the right-side, ensuring that their initial relative positions to each other remained.

*Meshes’ clean-up.* Manually reconstructing the cranium left two main gaps in the reconstructed templates’ surface meshes (cf. Supplementary Figure S5). The first gap appears in the frontal bone as a rupture on the right side, roughly starting at the maximum zygomatic arch (CZA) landmark level. This gap extends along the entire frontal bone, posteriorly reaching the level of porion before the parietal bone. It results from dividing the frontal bone into two separate segments to counteract the compression in the diploe described above.

The second gap is more prominent in the palate-adjusted model but still visible in the “wideNasal” reconstruction. This gap arises from the translation of the right maxillary fragment and the state of preservation in that area. The maxilla lacks the frontal process towards the *pars nasalis*, which, along with the frontal process of the zygomatic bone, should form a smooth circular contour that borders the orbital rim. This leaves the area incomplete compared to the more complete left side. We applied two distinct landmark-based approaches to fill these two gaps in both manual reconstructions.

For the frontal bone gap, we sampled two series of landmarks — 97 for the “wideNasal” template and 69 for the “widePalate”— along the edges, with landmarks placed on either side at roughly equal distances to the edges (Supplementary Figure S5). Based on these landmarks, a convex hull polygon surface mesh was calculated. We selected the convex hull topology instead of a Delaunay triangulation approach^35,59,60^ as it fills gaps uniformly, avoiding excesses or depressions while ensuring consistent height on both sides of the gap. Subsisting irregularities were removed via remeshing (Rvcg::vcgIsotropicRmeshing^61^) which recalculates the triangulation of the virtual surface according to defined structural properties, here to match the convex hull mesh resolution to the mean value of the virtual specimens’ surface model. The remeshed convex hull was then merged with the surface models of both manual reconstructions.

In contrast, for the nasal bone gap, the goal was not only to fill the gap left by moving the maxillary segment but also to recreate the circular contour, to reconstruct the missing frontal process of the right maxilla. To do so, we sampled three series of six landmarks along the longitudinal axis of the gap on the right maxillary bone. The first series, starting from at zygo-max superior (ZMU) landmark, followed the lower boundary of the right maxillary segment below the nasal area and it extended anteriorly to the nasal-premaxilla margin (NPM) landmark. The second series covered the length of the nasomaxillary suture along the maxilla’s proximal boundary. The third series, comprising eight landmarks, beginning at the right distal edge of the nasal segment, where it meets the frontal bone, and extending to the anterior part of the nasal segment. This series was sampled at a level before the nasal segment reached its maximum height.

Then, each series was transformed into 40 equidistant points (Supplementary Figure S5) along polynomial Bézier curves (bezier::pointsOnBezier^62^). Bézier curves are defined based on an interpolation function from control points, here the first and last landmark of each series, with the intermediate points defining the curvature of the spline^63^. Next, we inverted these new landmarks to create 40 pairs of three landmarks perpendicular to the nasal gap. Using these new points, again, we defined Bézier curves and sampled 70 evenly spaced landmarks along them.

Finally, we exported this new set of landmarks and processed them in AVIZO as a point cloud. We applied a Delaunay triangulation algorithm and further processed the rendered surface, following the same procedure used for the frontal bone gap. Combined, these protocols allowed us to recreate the missing contour and successfully fill the gaps in the nasal and frontal bone areas. The Delaunay triangulation algorithms achieve the tessellation of a set of points, satisfying the Delaunay Condition that no such point is part of the circumhypersphere of the triangles generated from the algorithm^64^.

*Sampling of landmarks.* We used the same approach of applying transformation values to the right-side segments to sample anatomical landmarks on the manually reconstructed DFN3-150 models. First, we sampled landmarks (Supplementary Table S2) from the original model. Next, we applied the transformation values used to adjust the right-side segments, as described above, to the corresponding landmarks of each reconstruction. We did this to ensure consistent placement and to minimize errors. We kept the landmarks on the left side the same across all reconstructed models. We achieved this consistency using the same coordinate system for both the original model and the reconstructions (“widePalate” and “wideNasal”).

### Virtual reconstructions

#### Retrodeformation after Schlager et al., 2018 protocol^41^.

We utilized the recently described surface registration protocol^65–67^ to acquire surface semilandmarks. Due to the deformation at the right cranial side, here the surface registration was a better fit for transferring a surface semilandmark patch from the left side than purely relying on more standard approached like thin-plate spline (TPS) warping^68^. Subsequently, we applied the retrodeformation protocol outlined by Schlager and colleagues (2018)^41^ (for a critique of this method, see also^69^) based on these semilandmarks.

##### Surface registration

The surface registration works best on single-layered meshes, which eliminates later mismatching of the iterative closest point (ICP) algorithm to internal or isolated surfaces^66,67^. Therefore, a single-layered triangular mesh of the original DFN3-150 specimen was extracted. The left viscerocranial aspect - extending from the frontozygomatic suture to the inferior alveolar margin and the zygomaticotemporal suture to the nasal-premaxilla margin (NPM) and zygo-max superior (ZMU) landmark (Supplementary Figure S6, S7) - was treated as reference a surface throughout the surface registration as this remained unchanged in the manual reconstructions. In contrast, the single-layered mesh counterparts of the right sides of the original and both manual reconstructions were considered as targets due to their non-identical positions. We then digitally cleaned and isotopically remeshed these meshes to ensure they have the same number of faces and resolution, improving the registration algorithm’s performance. On the left-side reference mesh, we sampled 120 surface semi-landmarks using a K-means clustering algorithm (Mopho::fastKmeans) on the mesh’s vertices.

Following these preparations, the reference mesh was mirrored and roughly aligned with each target mesh via TPS warping based on the previously collected “anchor” landmarks and 35 equidistant curve semilandmarks. The latter were digitized along three bilateral curves (Supplementary Figure S6, S7 and Supplementary Tables S2, S3) and slid following the minimized bending energy criterion^68,70^ to ensure bilateral homology between them.

Next, we applied an elastic Iterative Closest Point (ICP) algorithm to optimize the registration between the reference and target meshes. Here, elastic means the inclusion of a Gaussian smoothing displacement vector^66,67^ component. Once the vertices of both meshes were optimally aligned, we transferred the semilandmarks from the inverted left-side reference meshes to the right-side target meshes using a closest match approach (Morpho::transferPoint and Morpho::vert2points^61^). This process resulted in 240 surface semilandmarks (Supplementary Figure S7) covering the viscerocranium of each of the templates (original and the two manual reconstructions).

##### Retrodeformation

We applied the ‘taxonomy-free’ protocol developed by Schlager and colleagues^41^. This workflow, which uses the “closed-form solution” for symmetrizing bilaterally deformed objects as designed by Ghosh and colleagues^71^, retrodeforms a distorted specimen using bilateral symmetry^35,72^. Unlike the original implementation of this approach^46^, we included semilandmarks instead of relying solely on anatomically defined landmarks^68^. Sampling semilandmarks increases the coverage of the studied structure, addressing the absence of anatomical landmarks in incomplete specimens, a common challenge in paleontological studies. To apply this workflow, we sampled 34 bilateral anatomical landmarks and 88 semilandmarks along eight curves on the specimens. We also used the 240 surface semilandmarks (Supplementary Figure S6, S7 and Supplementary Table S2, S3). Thereby, symmetry was first calculated based on the landmark level (Morpho::retroDeform3d^61^) and then transferred to the surface model (Morpho::retroDeformMesh^61^).

#### Restoration after Amano et al., 2018 protocol^42^

To investigate the effect of different reconstruction methods on the study of DFN3-150 morphology, we also applied the restoration protocol developed by Amano and colleagues (2022)^42^. This protocol uses landmarks to describe the shape of specimens and their variation. The design aims to exploit taxonomically relative interspecific morphological variation as reference material. The distorted specimen is then projected perpendicular to a hyperplane that represents this variation^42^ in a principal component framework, which removes any deformation in the specimen, restoring it to match the reference crania hyperplane. We selected three female reference crania, two belonging to subadult individuals. These reference specimens were used to restore the shape of the DFN3-150 viscerocranium.

**Step 1**. We digitized a landmark set of 62 bilateral anatomical landmarks and 124 equidistant curve semilandmarks for the reference specimens, and the DFN3-150 templates (Supplementary Figure S6, S8; Supplementary Table S1, S2, S3). For the reference specimen, *Mandrillus sphinx* PRICT70, the missing landmarks on the P3 buccal cusps using a TPS algorithm were statistically reconstructed based on the other two reference crania. The same approach was used to calculate glabella and nasion for the ‘original’ template, and glabella, nasion and nasospinale for the ‘wideNasal’ and ‘widePalate’ templates (Morpho::fixLMtps^61^).

**Step 2.** To minimize morphological variation caused by structural asymmetries in the reference crania, we corrected deviations from symmetry using reflecting relabeling^68^ (Morpho::symmetrize^61^) and subsequent TPS warping of the meshes to the symmetrical landmark configurations (Morpho::tps3d^61^), creating a fully symmetrized shape for each specimen. Due to smaller displacement values this approach was favored over simply mirroring along a midsagittal plane. The remaining restoration workflow was applied with and without surface semilandmarks, resulting in two distinct triplets for the “original”, “wideNasal” and “widePalate” templates. Thereby, we account for the severity of the impact that reference crania could impose on the subsequent steps, as the restoration will follow their variation.

For the surface semilandmarks, a patch of 140 semilandmarks was created via K-means clustering (Morpho::fastKmeans^61^) of the mesh vertices of the left viscerocranial surface of the *Papio anubis* PRICT733 reference specimen. These semi landmarks were then mirrored to the right side using the midsagittal plane established from the previously reflected and relabeled anatomical landmarks. To prevent landmarks from being misplaced in the mesh interior, we used a script in R that inflates the landmarks along the surface normals and then projects them back on the surface (code kindly provided by Dr. Costantino Buzi and Prof. Antonio Profico).

The resulting surface semilandmark patch (280 points) was transferred to the rest of the comparative sample via TPS warping (Morpho::placePatch^61^). All in all, the landmark configuration without surface semilandmarks included a total of 186 anatomical landmarks and curve semilandmarks while for the second configuration the addition of 280 surface semilandmarks lead to a total of 466 points. In both cases, the first three principal component (PC) scores were used^42^, representing 100% of the reference interspecies variation.

### Error calculations

#### Surface registration

We evaluated intra-observer error in landmark placement affecting the preliminary step of the surface registration workflow (cf. Materials and Methods: I. Retrodeformation after Schlager et al., 2018 protocol^41^). Therefore, SK digitized all anatomical and curve semilandmarks three times in succession on the surface model of the “wideNasal” template over a period of three weeks.

Error was evaluated at each landmark position by first calculating Euclidean distances to the configuration centroid and then subtracting these distances from the corresponding ones of the mean configuration of the repetitions. The latter was extracted as differences in Euclidean Distances in mm and percentage (Supplementary Table S6).

To further quantify the effect of error in landmark placement onto the actual surface registration, we calculated the Euclidean distance of all vertices from each generated surface for each of the landmark configuration trials, comparing them against the target mesh surface (vertex to surface distance). We quantified the percentage of distances for three distance thresholds. We then calculated the standard deviation (Sd) of the Euclidean distances (Supplementary Table S7).

Next, we calculated the Riemannian Distance (RD)^33^ (also referred to as Procrustes distance; the geodesic distance between two shapes on the preshape space - a measure of shape proximity - the resulting space after partial GPA)^73^ of each trials’ transferred semilandmarks (Supplementary Table S7). The algorithm successfully registered the surfaces, with the main deflection observed at the boundaries of the area of application (Supplementary Figure S10), as reported in other studies^66,67^. Thus, sampling of “anchor” landmarks did not affect the acquisition of surface semilandmarks generated by the surface registration protocol.

### Measurement error for the statistical analysis

We calculated the effect of intra-observer error in landmark placement on the statistical analysis. However, here also the influence of digital mesh resolution in the comparative sample was factored. SK repeatedly sampled 33 anatomical landmarks on 3 specimens (Supplementary Tables S1, S2), each representing a decreasing resolution status, across 4 discrete time intervals: *t*_1_ = 0, *t*_2_ = 1 h after, *t*_3_ = 2 days after, and *t*_4_ = 7 days after.

We represented measurement error as the standard deviation (Sd) of the Euclidean distance (ED) of each landmark per species from its average^74^, accounting for four repetitions. To highlight inconsistencies among different approaches when calculating this index (ED), it was calculated based on raw landmark positions and Boas coordinates (Supplementary Figure S11). After an initial Procrustes registration, we multiplied the Procrustes shape variables by their centroid size measure to calculate Boas coordinates. This reversed the effect of scaling, returning the configurations to their original size while maintaining translation and rotation^75^. This new space approximates the conformation space^76^. Additionally, we calculated the pairwise Riemannian Distance (RD) for each species trial (Supplementary Table S11). Euclidean distances for most landmarks are kept below 1 mm Sd level, while the partial registration during the generation of Boas coordinates has not a major impact on this index. Notably, both zygo-max superior landmarks that were sampled on the lowest resolution surface, display the highest Sd value, exceeding the 2 mm threshold. For this, the sampling repetition at *t*_4_ appears to be the most variant, as indicative by the Riemanian Distance between repetitions. An Sd value of zygo-max superior exceeding the 1 mm threshold also applies to the highest resolution surface model, only to the left bilateral one landmark.

We assessed the repeatability of landmark placement using a MANOVA routine described as two-way Procrustes ANOVA^77^. In this setup, we treated landmark coordinates as dependent variables, with species and repetitions as factors. We calculated the relative repeatability as an Intraclass Correlation Coefficient (ICC) ranging from zero (0) to one (1)^74,78^, using the mean squares from the np-MANOVA. ICC quantifies the relative repeatability value of repeated measurements within individuals of different groups. Measurement error (ME) is then calculated as the percentage of the difference of the ICC value from 1 (no error). Before analysis, we applied partial Procrustes registration to all landmark configurations (Morpho::procSym^61^) and extracted their symmetrical component^79^. The subsequent regression was calculated with 10,000 permutations and type II Sum of Squares for residual randomization (RRPP::lm.rrpp.ws^80–84^).

The analysis of variance showed significant shape variability between species but not within sampling repetitions (Supplementary Table S8). The Rsq value was 0.98 (*p* = 0.0002), and a relative ME of 0.35%. We further confirmed the insignificance of the trial effect on specimens’ shape using a blocked permutational MANOVA. We restricted the 10,000 permutations in the species factor, accounting for non-independence (non-exchangeable terms) within trials and using type I Sum of Squares (vegan::adonis2^85^), which resulted in a p-value of *p* = 0.97 for the repetitions (Supplementary Table S9).

For both permutational approaches, we tested landmark configurations per species and trial for multivariate homogeneity of variance (vegan::betaspider and vegan::permutest with 10,000 permutations^85^) and found it well above the acceptable levels of α≤0.05 (Supplementary Table S10). These additional steps in error measurement assessment were crucial due to the absence of a standardized threshold during the evaluation of measurement error regardless of trial or mesh resolution.

### Statistical analysis

#### Data Processing

Given the deterministic nature of the reconstructions and the associated errors that are introduced, we decided to investigate the viscerocranium affinities of DFN3-150 using only the 33 anatomical landmarks (Supplementary Figure S6; Supplementary Table S2) sampled on facial bones and the rostral part of the frontal bone. Those landmarks refer to Type I and II except for frontomalare temporale that is a Type III landmark^86^. Due to missing bone surfaces, we estimated nasospinale and prosthion landmarks in the DFN3-150 retrodeformed models using a TPS algorithm from the remaining comparative material, with the exception of nasospinale for the two original fossil models restored based on Amano and colleagues’ (2022) restoration protocol^42^. In the subsequent workflow described below, we did not allow these calculated landmarks to slide.

Several anatomical landmarks (see Supplementary Table S2) were allowed to slide (see^87^) like surface semilandmarks, minimizing a bending energy criterion^88^, to compensate for uncertainties in their placement due to low mesh resolution and to secure identification of some features, like closed sutures. After extracting the adjusted landmarks, we symmetrized all comparative sample configurations using a reflected relabeling procedure^68^ (Morpho::symmetrize^61^). These landmark configurations, serve as the primary variables used in this study. Immediately after, we transformed all the respective meshes of the comparative material, to adjust them to the symmetrized landmark configurations (Morpho::tps3d^61^). Those configurations and meshes, alongside to the initial slidden DFN3-150 models’ landmark configurations, will serve as the primary form variables for this study.

To assess whether the number of landmarks was sufficient to capture morphological variation among samples, we applied the LaMBDA::lasec function^89^ (Supplementary Figure S12) which quantifies the contribution to variation of a sequentially increasing number of landmarks, as opposed to the full configuration by calculating each time the Procrustes Sum of Squares (PSS) between this subset and the full configuration. For the present sample a good fit is reached already at 30 landmarks. Landmark configurations of the comparative material were initially subjected to a partial Procrustes registration alignment (GPA), which scales specimen to unit centroid size and removes differences in position and orientation (for a discussion see^73,90^). Centroid size of a landmark configuration is calculated as the square root of the sum of squared distances of all landmarks from their centroid^73,76^. Afterwards we calculated the centroid size (Morpho::Csize^61^) for all DFN3-150 models and aligned them with the comparative sample GPA (Morpho::align2procSym^61^) allowing the comparative sample not to be influenced by the inclusion of the fossil specimens in calculating their mean, and so resulting in an unbiased reference sample. Finally, Procrustes coordinates were projected in the shape tangent space, a linear approximation of the Kendall’s shape space – with the correlation^61^ of the coordinates between the two spaces around 0.999 (Morpho::regdist^61^).

#### Shape variation and allometry

We submitted the tangent space coordinates to a shape space principal component analysis (PCA), in which fossil specimens were projected onto the principal component space^40^. After assessing the number of meaningful PC scores (Morpho::getMeaningfulPCs^61^), we performed a paired, two-tailed nonparametric Kendalls’ tau (τ)^44^ (stats::cor.test^91^) to investigate whether allometric effects are integrated in the observed separation along these PC axes. Through this test, we examined the association between each species’ first three PC scores and the species’ centroid size. A non-parametric test was necessary due to the absence of multivariate normal distributions for the first two PC scores (74.33% cumulative proportion) and the centroid size (MVN::mvn^92^ bootstrap ‘energy distance’ multivariate normality test: E-statistic: 1.27; *p* < 0.05). Before analyzing allometric effects, we tested the shape variable per genus group for multivariate homogeneity of variance (group dispersion) with 10,000 permutations and a spatial median type of analysis (vegan::betaspider and vegan::permutest^85^).

Additionally, we assessed whether size was independent of genus by performing a Wilcoxon-Mann-Whitney non-parametric test^93^ (coin::wilcox_test function^93^ with a Monte Carlo approximation based on 10,000 iterations), indicating non-independence of size in respect to genus, guiding us towards the selection between type II and III Sum of Squares choice for the allometric regressions. We further assessed the magnitude of their association as an effect size, using the Wilcoxon test (rstatix::kruskal_effsize^94^), which yielded a large value. This non-parametric test was preferred because the assumption of homogeneity of variances for size between the two groups was not met, as indicated by the Barletts’ test (stats::barlett.test^91^), while the normality assumption, explored via Shapiro-Wilk test (stats::shapiro.test^91^), was not violated for both genera (Supplementary Table S14).

To assess the statistical significance of evolutionary allometry, we then performed a series of multivariate regressions of shape on size and genus (RRPP::lm.rrpp.ws^80–84^ with 10,000 permutations). This function accommodates hierarchical data structures by explicitly modeling within-subject (here within species) variation. First, a static allometry model (shape ~ size) was fitted with Type I Sum of Squares. Next, we fitted a common allometry model with both centroid size and genus as predictors (shape ~ size + genus), applying Type II sums of squares, which is recommended for unbalanced designs with correlated predictors and no significant interaction^95^. Finally, we conducted a unique allometry regression that included the interaction term (shape ~ size * genus), evaluated using Type III sums of squares to appropriately partition variance in the presence of interactions in unbalanced data^95,96^. For comparative purposes of the significance of the predictors in the allometric regressions, we conducted a MANCOVA (RRPP::lm.rrpp.ws^80–84^) on the comparative material within a phylogenetic context, using a phylogenetic tree topology as the covariance matrix. As a reference, we used a cladogram based on the consensus chronogram for 12 species across two genera included in this study. This consensus follows a Bayesian phylogeny model obtained from the 10kTrees project^97^ website (Supplementary Figure S13; https://10ktrees.nunn-lab.org/index.html). In order to examine collinearity among predictors (a measure of their overlap), we use the Variance Inflation Factor (VIF) and tolerance, which is the reciprocal of VIF^98,99^.

Due to the limited number of *Papio* specimens, we evaluated the robustness of the common allometry regression results by performing a rarefaction test for the unique allometry model, in which we assessed the similarity (homogeneity) of angles^100^ using the statistical significance (F-statistic) of the interaction term under rarefaction. The R code for this analysis was adapted from Ariel Marcy^101^. We applied the test in both ordinary least squares (OLS) and generalized least squares (GLS) regression models, with the phylogenetic covariance matrix. Effectively, we examine whether genera follow their own shape-to-size relationship (different slopes) under balanced sample sizes. In that sense, rarefaction is a form of undersampling. For this, we performed 1,000 rarefaction iterations by randomly selecting 7 *Macaca* (to match the *Papio* sample size), combining them with all *Papio* specimens, and recalculated the unique allometry regression. For each rarefied set, we also generated a null distribution by shuffling genus labels among these 14 rarefied specimens and recalculated the unique allometry regression for 20 iterations (blocked in each rarified iteration). Thereby, we artificially destroyed the biological meaningfulness of the dataset that links genera to size and shape. This approach allowed us to directly evaluate whether observed genus differences in allometric slopes could be explained solely by sampling effects, and, at the same time, whether this holds under a complete disruption of the correspondence between genus and size and shape.

While we could use the RRPP::pairwise^80–83^ function to assess the correlation of slopes, such a choice is redundant in the presence of only two groups in our analysis, but, due to methodological limitations^82,83^, we would not be able to apply the same test for the phylogenetic regression. For both regressions we report the resulting p-values and Z-scores for the F statistic. The Z-score, as a measure of effect size, expresses standard deviations of the observed F-statistic from a distribution generated with residual permutation with the RRPP package^83,102,103^.

To further disentangle the signal of size from genus-related differences in cranial shape, we modeled and then predicted the shape variables as a function of genus and a constant centroid size, corresponding to the mean centroid size of the complete comparative sample. This allowed us to control for allometric effects while assessing genus-level shape variation. The predicted mean shape for each genus, along with its associated 95% confidence intervals, was projected onto the principal component axes of shape-space. This approach enables the assessment of genus-specific shape affinities while holding size constant, thereby clarifying taxonomic distinctions that are not attributable to size-related allometric variation. For this, we used the stats::predict function^91^.

Overall, allometric relationships in this analysis were visualized using prediction lines and regression scores^104,105^. Regression scores represent “the shape variable associated with the shape changes predicted by the regression model, but also includes the residual variation in that direction of shape space” (^104^; p. 72). To visually present the variation expressed at each landmark as well as the direction of change, warped meshes were generated, depending on the underlying analysis via landvR::coordinates.difference^106^, landvR::Procrustes.var.plot^106^ functions, geomorph::shape.predictor^80–83^ and Morpho::tps3d^61^.

#### Software

Segmentation, data collection and the Delaunay triangulation were held in Avizo 8.0.0, Avizo 9.2.0 Lite and AVIZO 3D 2023.1.1 software (©FEI Visualization Sciences Group). Figures were generated via rgl (v1.3.31) and ggplo2 (v4.0.1) packages^91^ ad. Surface semi-landmarks were sampled with the fastKmeans function and curve semi-landmarks were made equidistant via equidistantCurve from Morpho^61^ (v2.13) package. Those semilandmarks were sliden using the relaxLM and slider3d functions from Morpho. Meshes’ cleanup was held by vcgIsotropicRemeshing function from Rvcg^61^ package. Points on Bezier curves were sampled via pointOnBezier function of the Bezier^62^ (v1.1.2) package. For the surface registration step we used vert2points, fastKmeans and transferPoints functions from Morpho, alongside gaussMatch from mesheR^107^ (v0.4.200213). Retrodeformation of the DFN3-150 models was done using retroDeform3d and retroDeformMesh functions from Morpho, while during the computerized restoration, we used the fixLMtps, symmetrize, tps3d and placePatch functions from Morpho. For data processing we used procSym, Csize and alignProcSym functions from Morpho and lasec from the LaMBDA^89^ (v0.1.10000) package. For the overall display of the data, we used coordinates.difference and Procrustes.var.plot functions from the landvR^106^ (v0.5.3) package, shape.predictor from the geomorph^80–83^ package (v4.0.10) and tps3d from Morpho. Preparatory statistics for the study allometry were calculated with the getMeaningfulPCs function from Morpho, cor.test, barlett.test, shapiro.test functions from the stats^91^ (v4.5.1) package, and mvn, wilcox_test and krustal_effesize functions from MVN^92^ (v6.2), coin^93^ (v1.4-3) and rstatix^94^ (v0.7.3) packages respectively. For the allometric regressions framework, we used the lm.rrpp.ws and pairwise functions from RRPP^80–83^ package (v2.1.2.999), and for shape prediction the predict function (stats). Measurement error for the statistical analysis was evaluated via lm.rrpp.ws function from RRPP, and the adonis2, betaspider and permutest functions from vegan^85^ (v2-7.2).

## Supplementary Information

Below is the link to the electronic supplementary material.

Supplementary Material 1

Supplementary Material 2

## Data Availability

The data of this study are available upon reasonable request. R scripts for the analysis is available for non-commercial use at Github. *Paradolichopithecus* aff. *arvernensis* DFN3-150 micro-CT slices are available from their respective housing institution/repository upon reasonable request.
