# Supplementary material for: Virtual reconstruction and analysis of the face of DFN3-150 Paradolichopithecus aff. arvernensis specimen from Dafnero, Greece
Source: Sci Rep. 2026 May 10;16:14703. doi: 10.1038/s41598-026-51595-8 (PMC13158299; doi:10.1038/s41598-026-51595-8)
Supplement: Supplementary file 1 — Supplementary Material 1 [file 41598_2026_51595_MOESM1_ESM.docx]

**Virtual reconstruction and analysis of the face of DFN3-150 *Paradolichopithecus* aff. *arvernensis* specimen from Dafnero, Greece.**

Stylianos Koutalis, Carolin Röding, Gildas Merceron, Franck Guy, Dimitris S. Kostopoulos, Katerina Harvati

**Table of contents**

[Supplementary Figures 3](#_Toc226025406)

[Figure S1 3](#_Toc226025407)

[Figure S2 4](#_Toc226025408)

[Figure S3 4](#_Toc226025409)

[Figure S4. 5](#_Toc226025410)

[Figure S5 6](#_Toc226025411)

[Figure S6 7](#_Toc226025412)

[Figure S7 8](#_Toc226025413)

[Figure S8 9](#_Toc226025414)

[Figure S9 10](#_Toc226025415)

[Figure S10 10](#_Toc226025416)

[Figure S11 11](#_Toc226025417)

[Figure S12 12](#_Toc226025418)

[Figure S13 12](#_Toc226025419)

[Figure S14 13](#_Toc226025420)

[Figure S15 14](#_Toc226025421)

[Figure S16 15](#_Toc226025422)

[Figure S17 16](#_Toc226025423)

[Figure S18 17](#_Toc226025424)

[Figure S19 18](#_Toc226025425)

[Figure S20 19](#_Toc226025426)

[Figure S21 20](#_Toc226025427)

[Supplementary Tables 21](#_Toc226025428)

[Table S1 21](#_Toc226025429)

[Table S2 23](#_Toc226025430)

[Table S3 24](#_Toc226025431)

[Table S4 24](#_Toc226025432)

[Table S5 25](#_Toc226025433)

[Table S6 25](#_Toc226025434)

[Table S7 26](#_Toc226025435)

[Table S8 26](#_Toc226025436)

[Table S9 26](#_Toc226025437)

[Table S10 26](#_Toc226025438)

[Table S11 27](#_Toc226025439)

[Table S12 27](#_Toc226025440)

[Table S13 27](#_Toc226025441)

[Table S14 28](#_Toc226025442)

[Table S15 28](#_Toc226025443)

[Table S16 28](#_Toc226025444)

[Table S17 29](#_Toc226025445)

[Table S18 29](#_Toc226025446)

[References 30](#_Toc226025447)

# Supplementary Figures

**
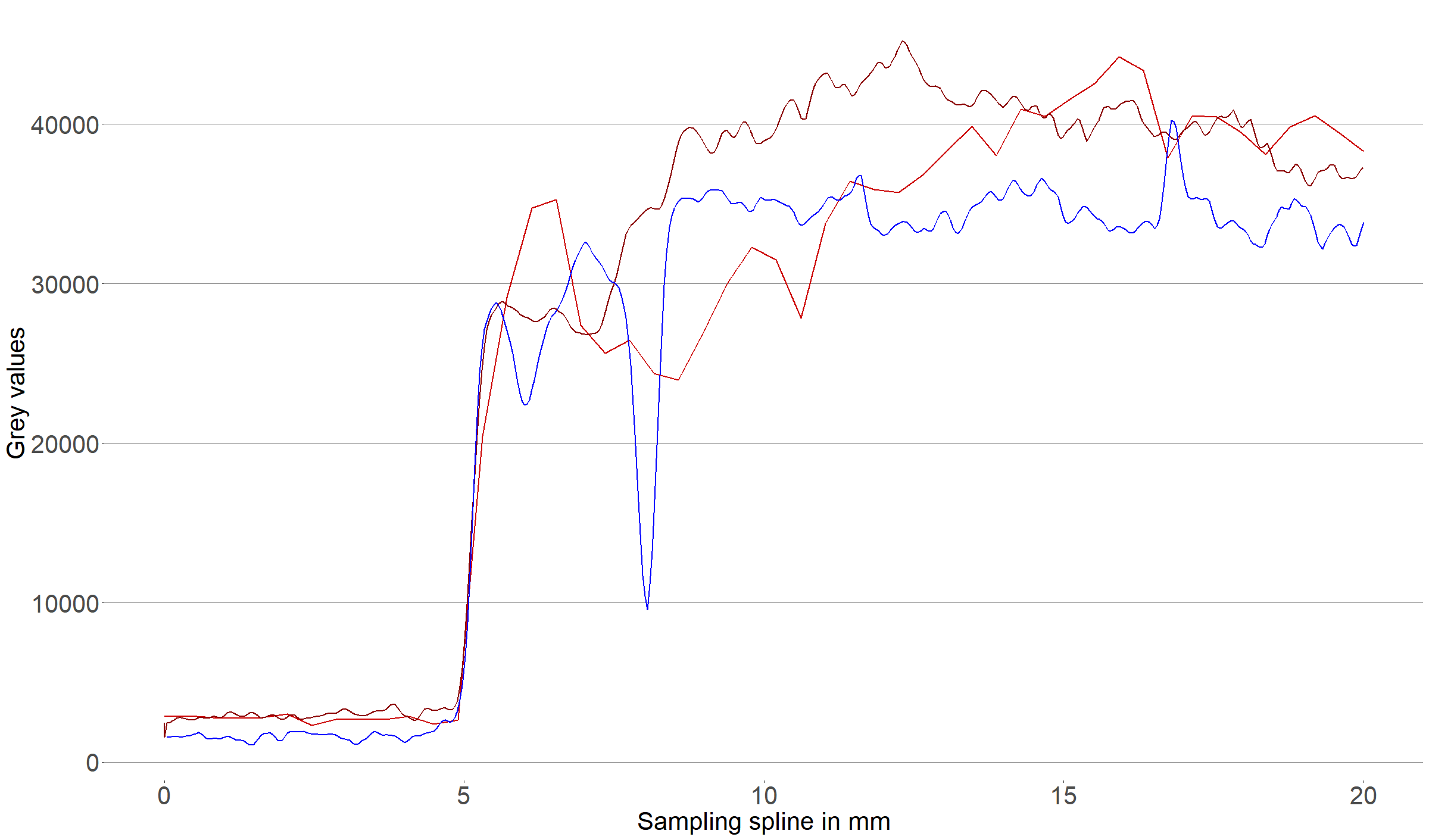
**

Figure S1**.** Grey value ranges for three distinct types of bone to matrix visual representation differences for DFN3-150^1^. In a 16-bits unsigned image, materials are described with (2^16=) 65256 different grey values, with 0 denoted as black (i.e. air) and 65255 as white (i.e. enamel-the densest materials). Sampling was applied in the neurocranium for a 15 mm spline, starting from the outer table of the bone (0-5cm; outer air, 5 to ~8mm; bone, 8 to 20mm: inner matrix. The thinner and shallower the curves’ depression gets for the transition from bone to inner matrix, the more difficult the discrimination between them is. Different colors display different parts of the neurocranium sampled. The same conditions can be extrapolated for the viscerocranium of DFN3-150.

**
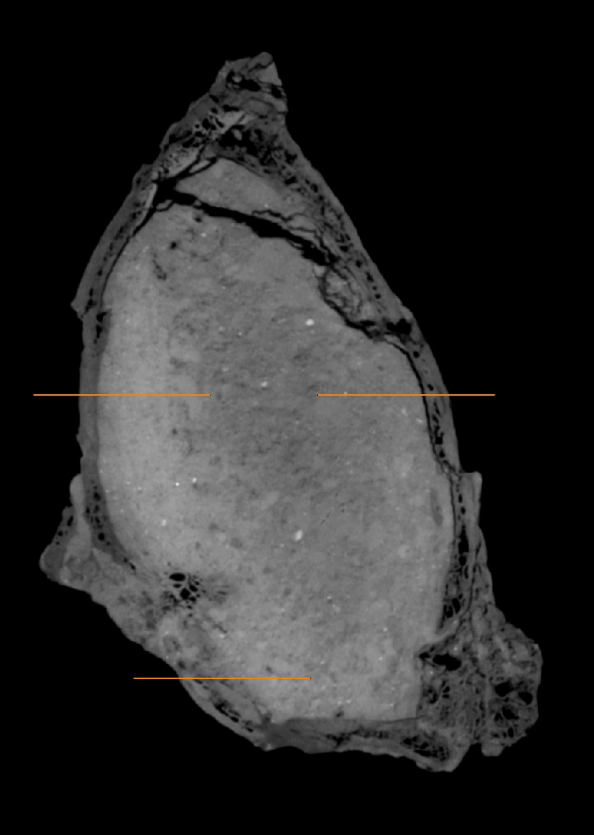
**

Figure S2**.** Exact positions of where the splines were sampled. The upper right spline represents the blue curve in Figure S1, the upper left the brown curve, and the bottom right, the red curve.


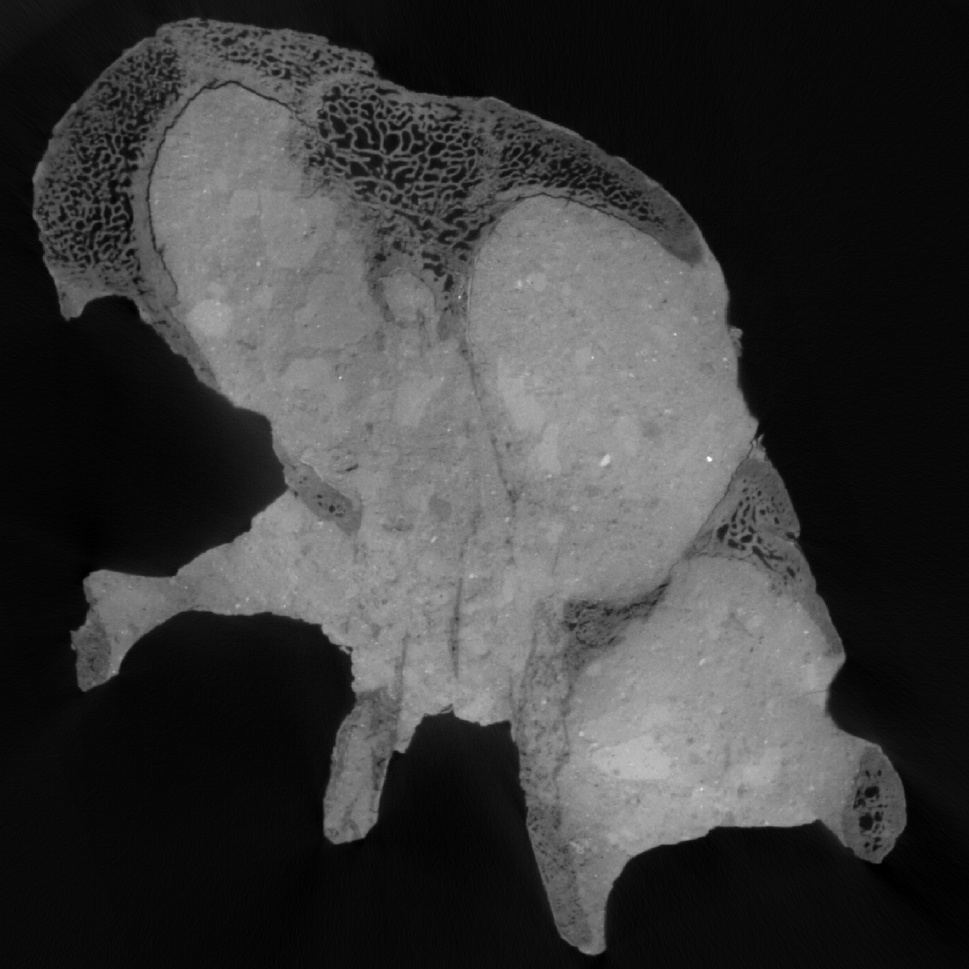


Figure S3**.** The rostral part of the frontal bone with the characteristic triple structure. Frontal view. Unscaled. Modified from[[1](#_ENREF_1)]


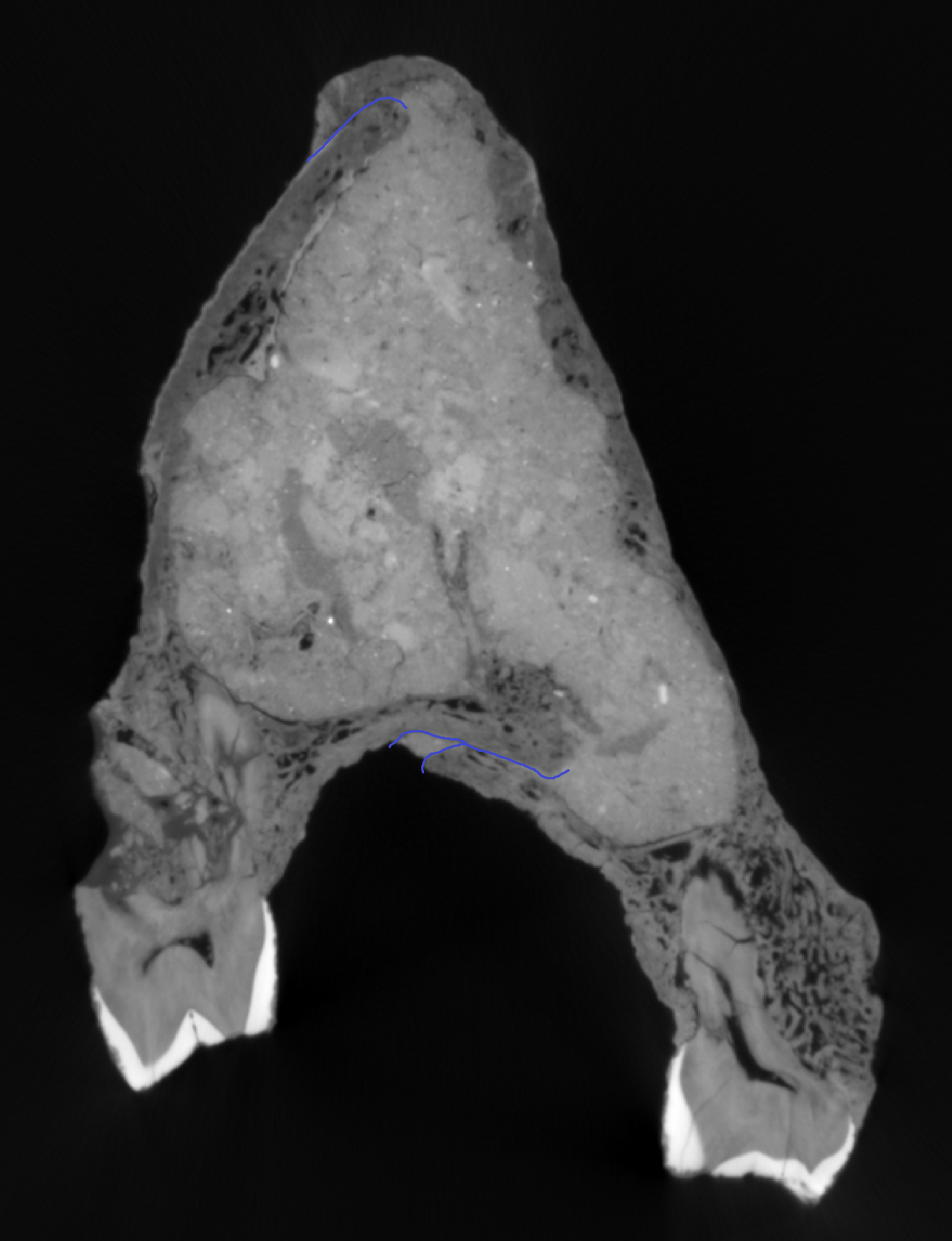


Figure S4. The palatal and nasal overlap. Boundaries are delimitated with blue line. Frontal view. Unscaled.


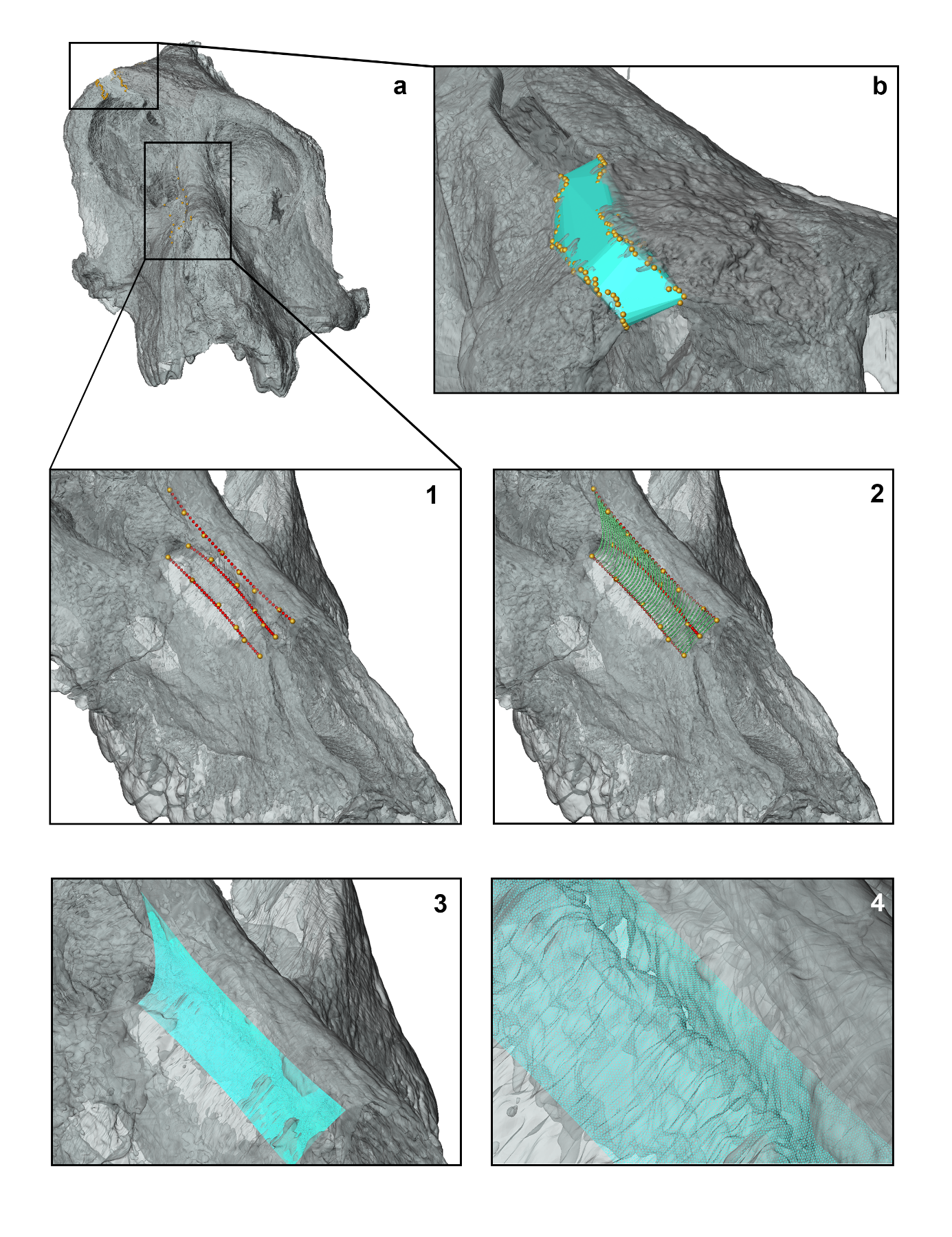


Figure S5**.** Schematic workflow for filling the gaps after manual reconstruction: In a) the DFN3-150 ‘wideNasal’ template is shown with two regions where landmarks were sampled along the gaps. In b), the convex hull of the landmarks sampled on the frontal bone gap is displayed. In 1 and 2 are the individual steps of the application using points sampled along Bezier curves. In 3 and 4 is the final mesh generated after the Delauny triangulation of the resulting points. Detail boxes depict the specimen in right lateral oblique view, while box a) presents the frontal view.


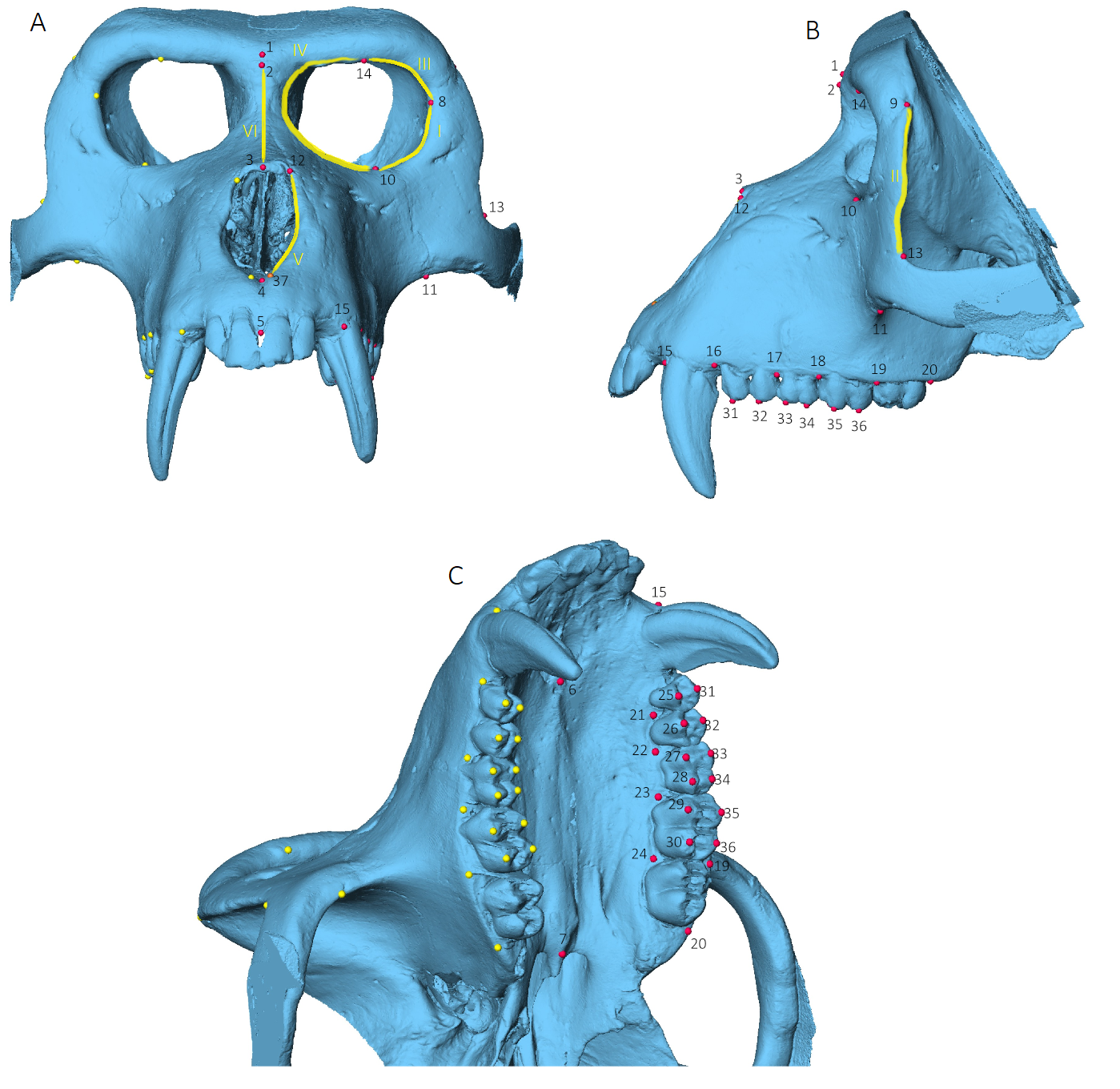


Figure S6. Total configuration of anatomical landmarks. Landmarks on the left side are shown in red and are indexed, while yellow indicates their right counterparts (for landmark definitions see Supplementary Table S2). The ‘ePA’ landmark, used solely in *Paradolichopithecus* reconstructions is shown in orange. Outlines correspond to curve semilandmarks (Supplementary Table S2). In ***A***) frontal; ***B***) left lateral and ***C***) oblique ventral views. Figures not to scale.

**
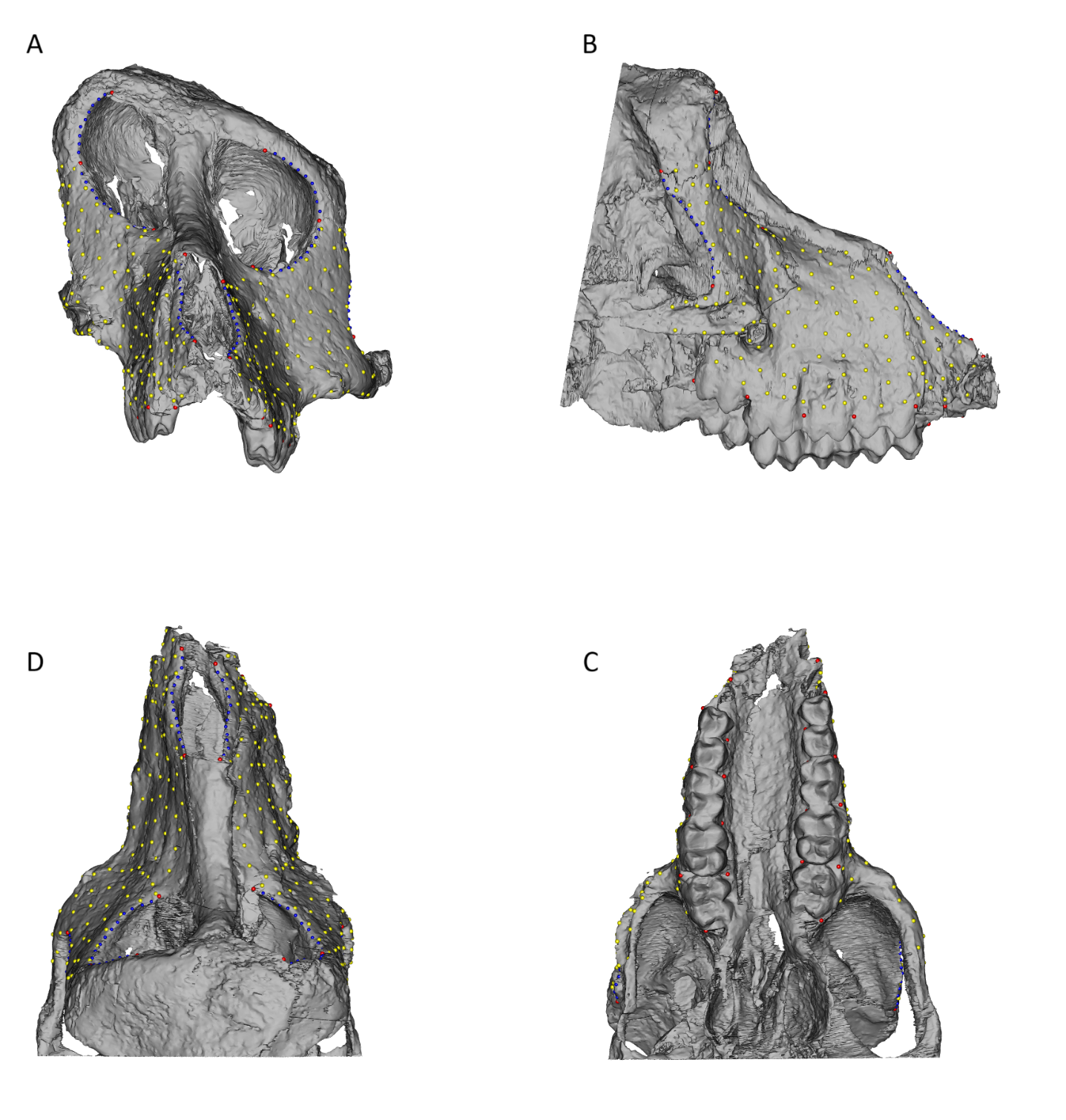
**

Figure S7**.** The original *Paradolichopithecus* aff. *arvernensis* DFN3-150 with the landmark configuration used for the Schlager et al. (2018) retrodeformation protocol[[2](#_ENREF_2)]. In red are depicted the anatomical–fixed landmarks, in blue the semilandmarks sampled in predefined curves, and in yellow the surface semilandmarks (for landmark definitions see Supplementary Table S2). **A)** frontal view; **B)** right lateral view; **C)** dorsal view; and **D)** ventral view. Figures not to scale.


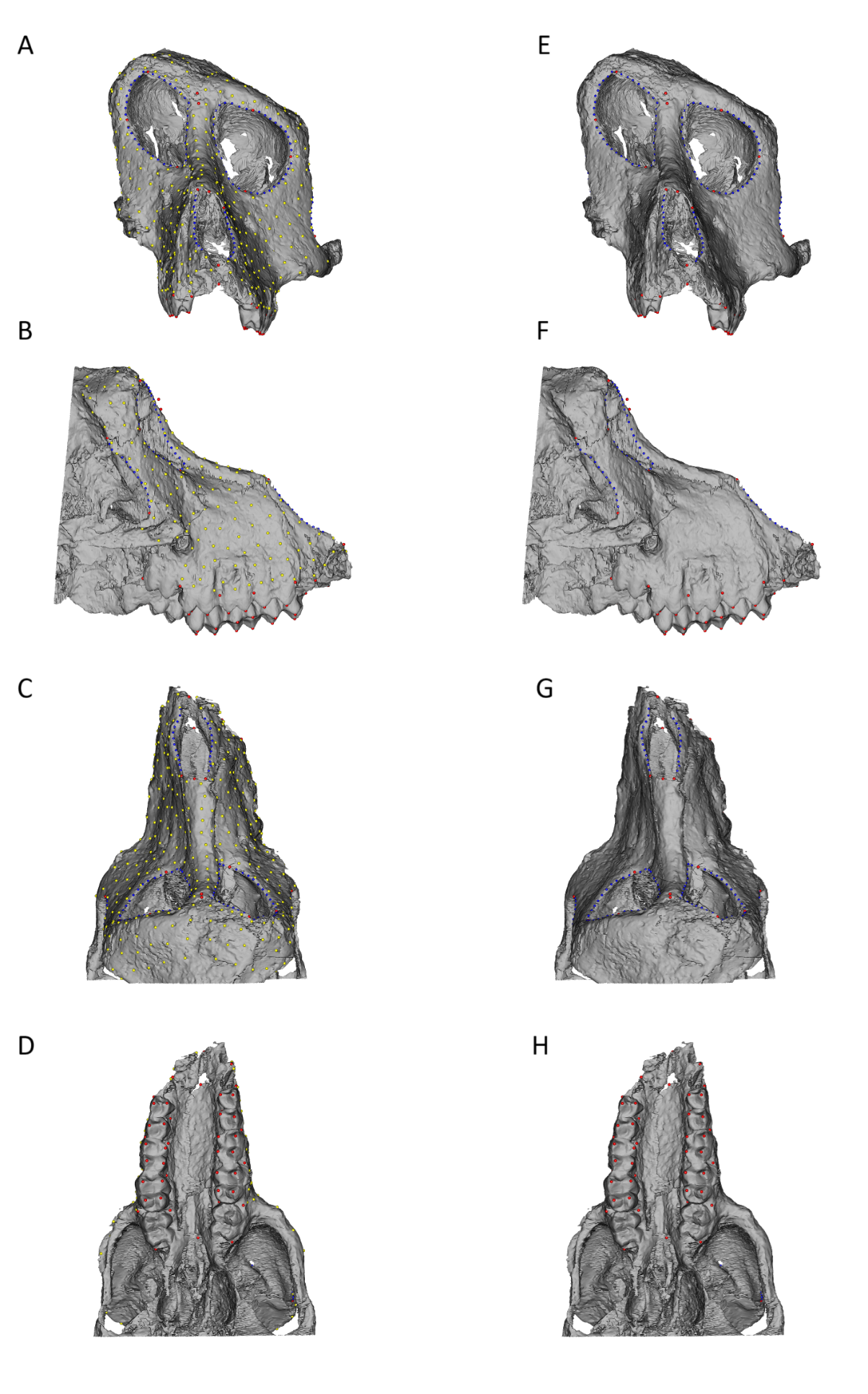


Figure S8**.** The original *Paradolichopithecus* aff. *arvernensis* DFN3-150 with the landmark configuration used for the Amano et al. (2022) restoration protocol[[3](#_ENREF_3)]. Red depicts the anatomical-fixed landmarks; blue depicts the semilandmarks sampled in predefined curves; and yellow depicts the surface semilandmarks (for landmark definitions see Supplementary Table S2). **A-D)** represents the configuration used for the restoration with surface semilandmarks in frontal, right lateral, dorsal and ventral views; **E-F**) the same configuration used for the restoration without surface semilandmarks in frontal, right lateral, dorsal and ventral views. Figures not to scale.


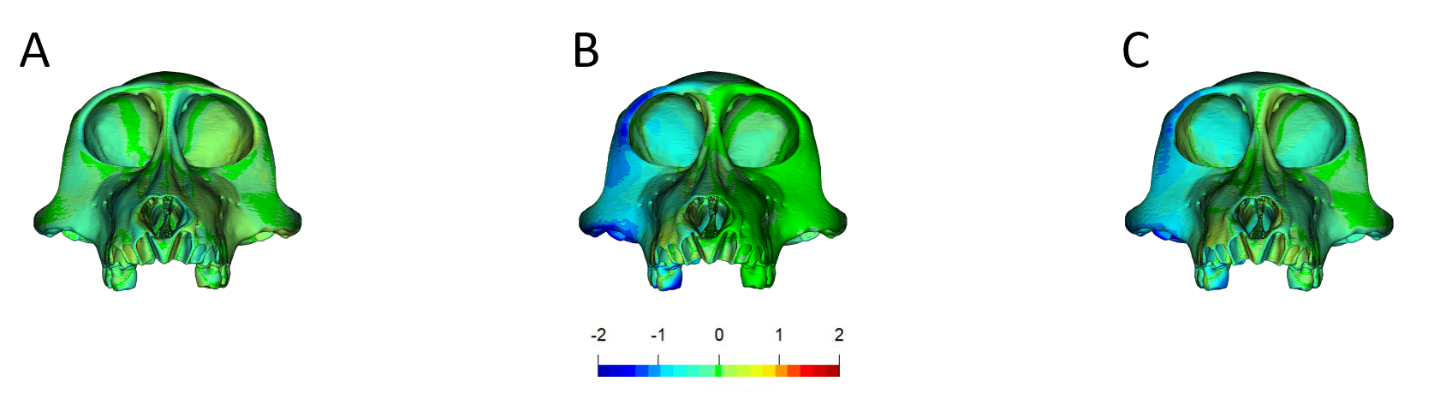


Figure S9. Surface displacement maps (Morpho::meshDist)[[4](#_ENREF_4)] for the Amano et al. (2022) protocol[[3](#_ENREF_3)] on *Mandrillus sphinx* PRICT70 reference cranium. The meshes generated from the **A)** reflected relabeled and **B)** mirrored configurations are depicted onto the original specimens’ mesh, as well as **C)** those between them. Colors depict distances. Red indicates a maximum surface excursion of 5 mm and blue a maximum surface depression of 2 mm in relation to the reference mesh. Color scale threshold is set to 0.05mm.


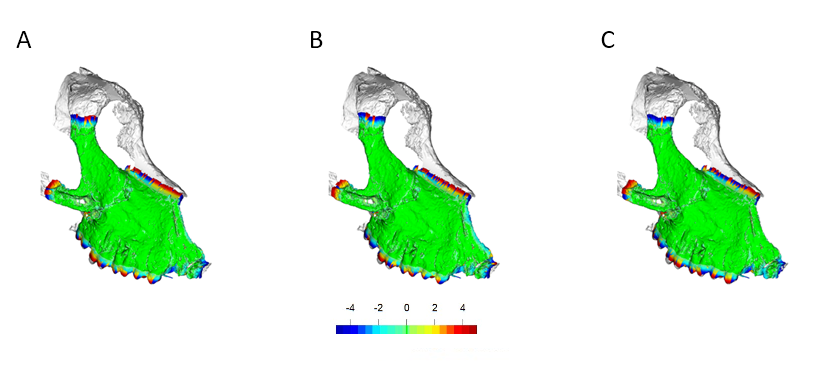


Figure S10. Surface displacement maps (Morpho::meshDist)[[4](#_ENREF_4)] for three successive (**A**; **B**; **C**) of the registered target surface for each ‘anchor’ landmarks trial, during for the initial alignment step in the surface registration algorithm. The ‘wideNasal’ model is depicted. Red represents a maximum surface excursion of 5 mm and blue a maximum surface depression of 5 mm in relation to the reference mesh. Color scale threshold is set to 0.05mm.

**
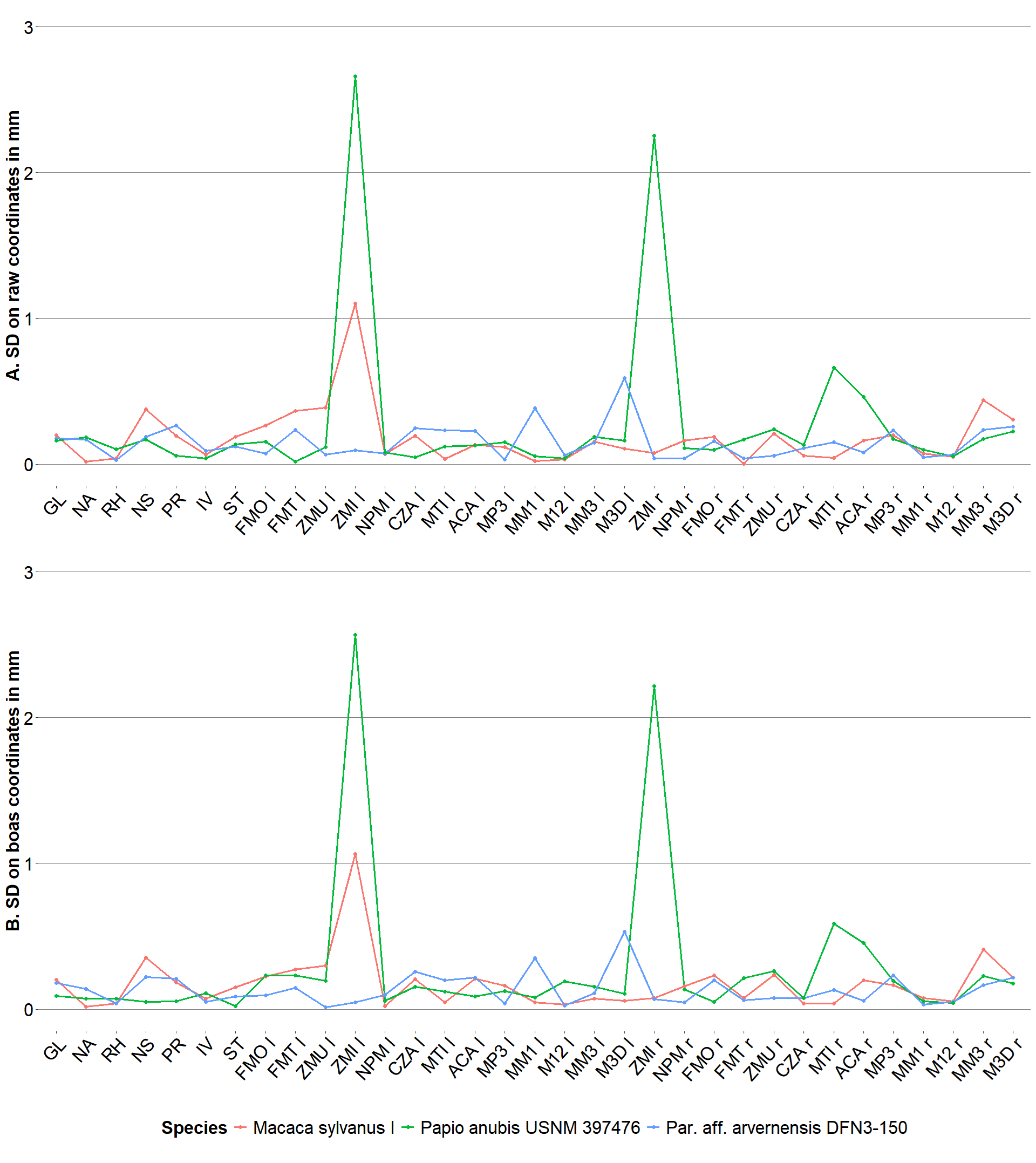
**

Figure S11. Histograms of the resulting standard deviation of each landmark per species, using superimposed coordinates depicting sampling precision. **A)** using the raw coordinates and **B)** using Boas coordinates. Units are in mm.

**
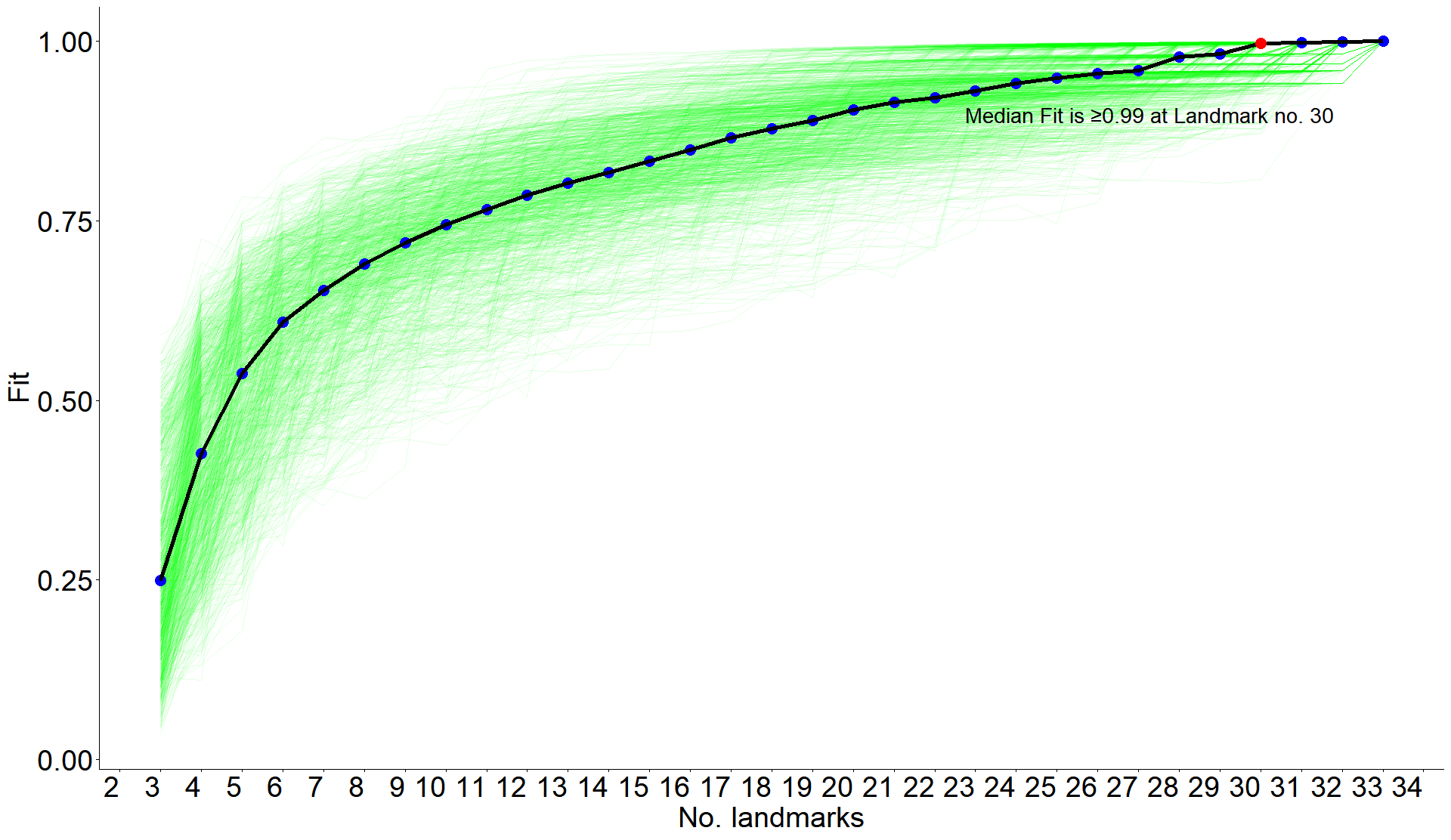
**

Figure S12**.** Sampling curve after LaSEC application[[5](#_ENREF_5)] for a parent landmark dataset (33) used in the comparative analysis. Median fit (blue curve), an indicator of shape robustness, reaches and exceeds the 0.99 value at 30 landmarks.


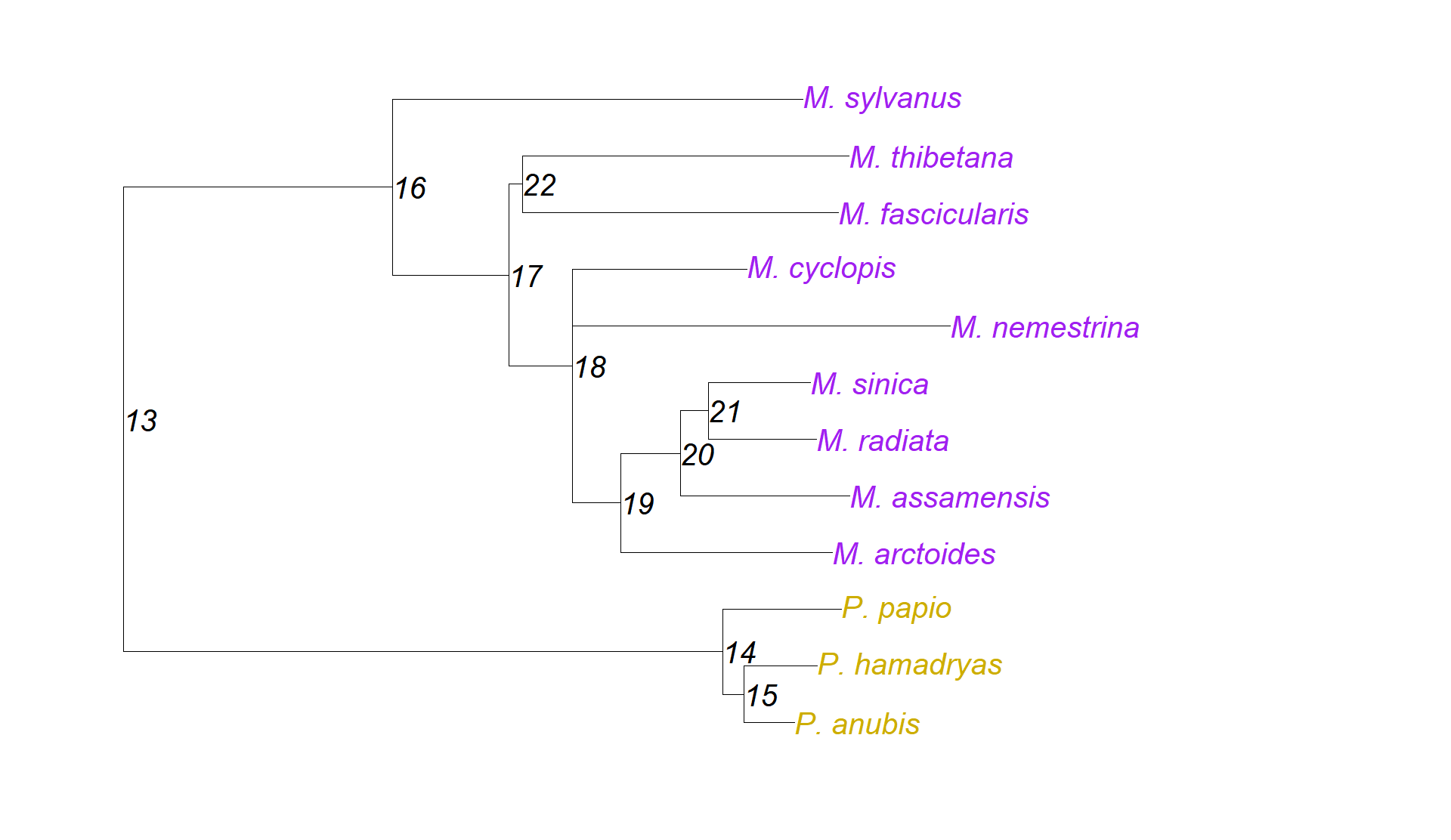


Figure S13**.** The consensus phylogenetic tree used in the study. Downloaded from 10kTrees project[[6](#_ENREF_6)] website (https://10ktrees.nunn-lab.org/index.html). In purple *Macaca* and orange, *Papio* species.


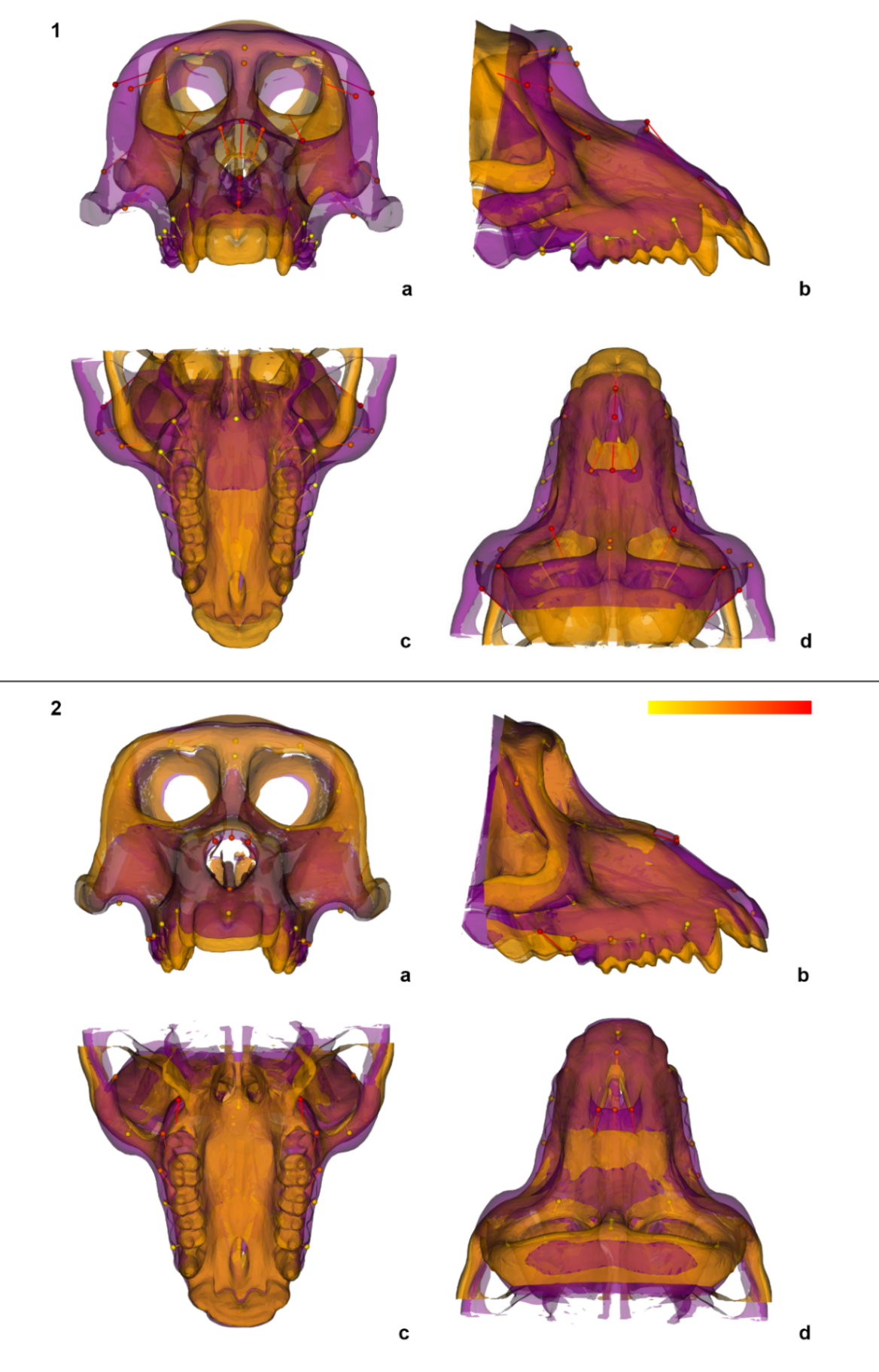


Figure S14. Shape changes associated with the PCA plot in Figure 2 in the main text. The upper part (1) refers to the shape changes in PC1 and (2) in PC2; a) frontal, b) right lateral, c) ventral, and d) dorsal views. Green meshes depict the predicted shape in the minimum of each axis, and purple the maximum. Overlaid are the respective landmarks with the vectors of change. The color scale depicts the magnitude of variation of each landmark.


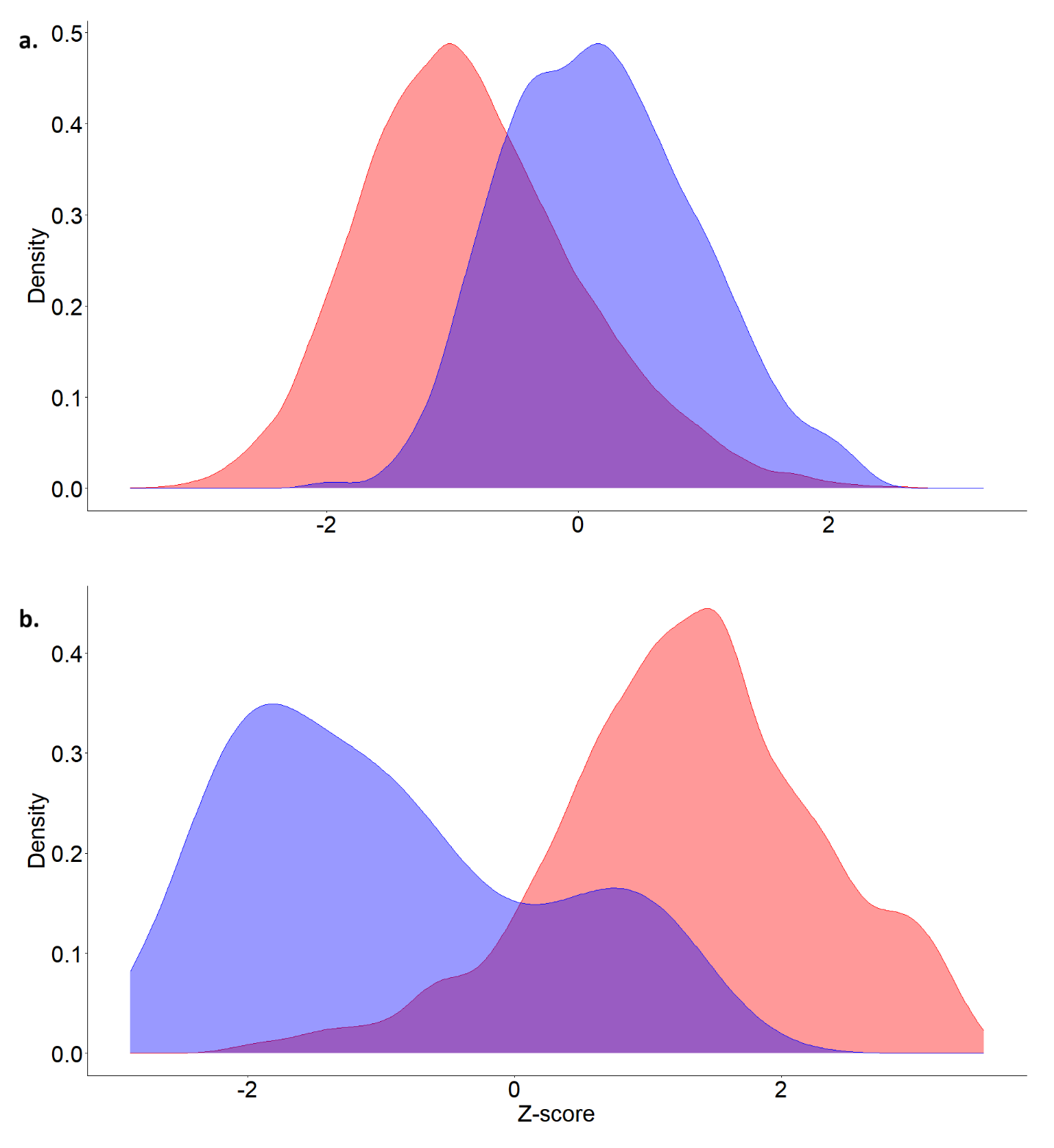


Figure S15. Density plots of the rarefied results of the Z-score effect sizes for the (a) OLS and (b) the GLS regressions. The null rarefied distribution is displayed in red, and in blue the rarefied.


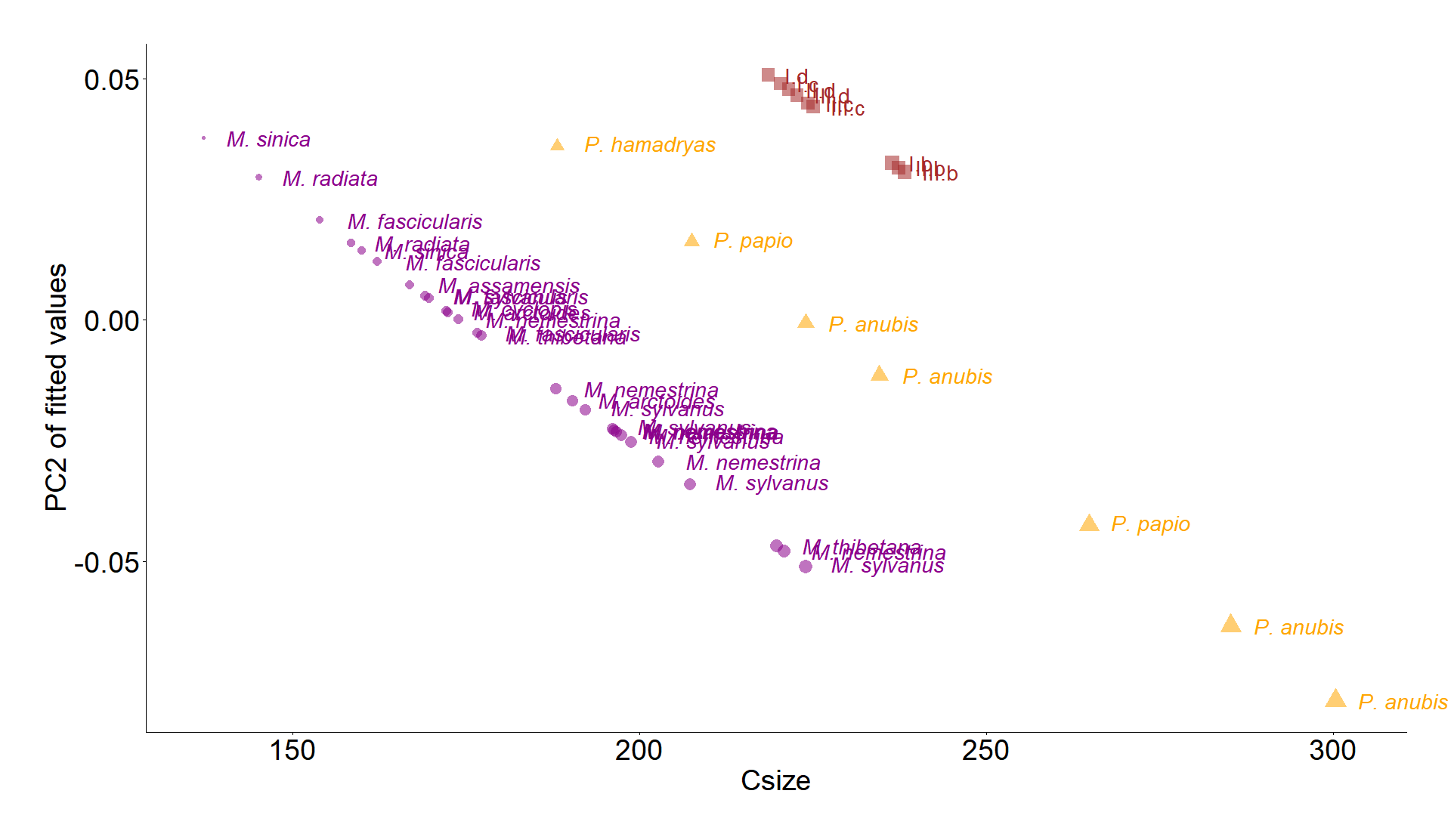


Figure S16. PC2 of fitted values of the common allometry regression plotted against centroid size. In purple are *Macaca*, orange are *Papio*, and in red are *Paradolichopithecus* models. Centroid size differences are graphically displayed with the symbol’s size. *Paradolichopithecus* abbreviations follow Figure 1 of the main text numeration; letters, b, c, d, correspond to the virtual reconstruction protocol and Latin numbers I, II, III correspond to the template upon which these protocols were applied.


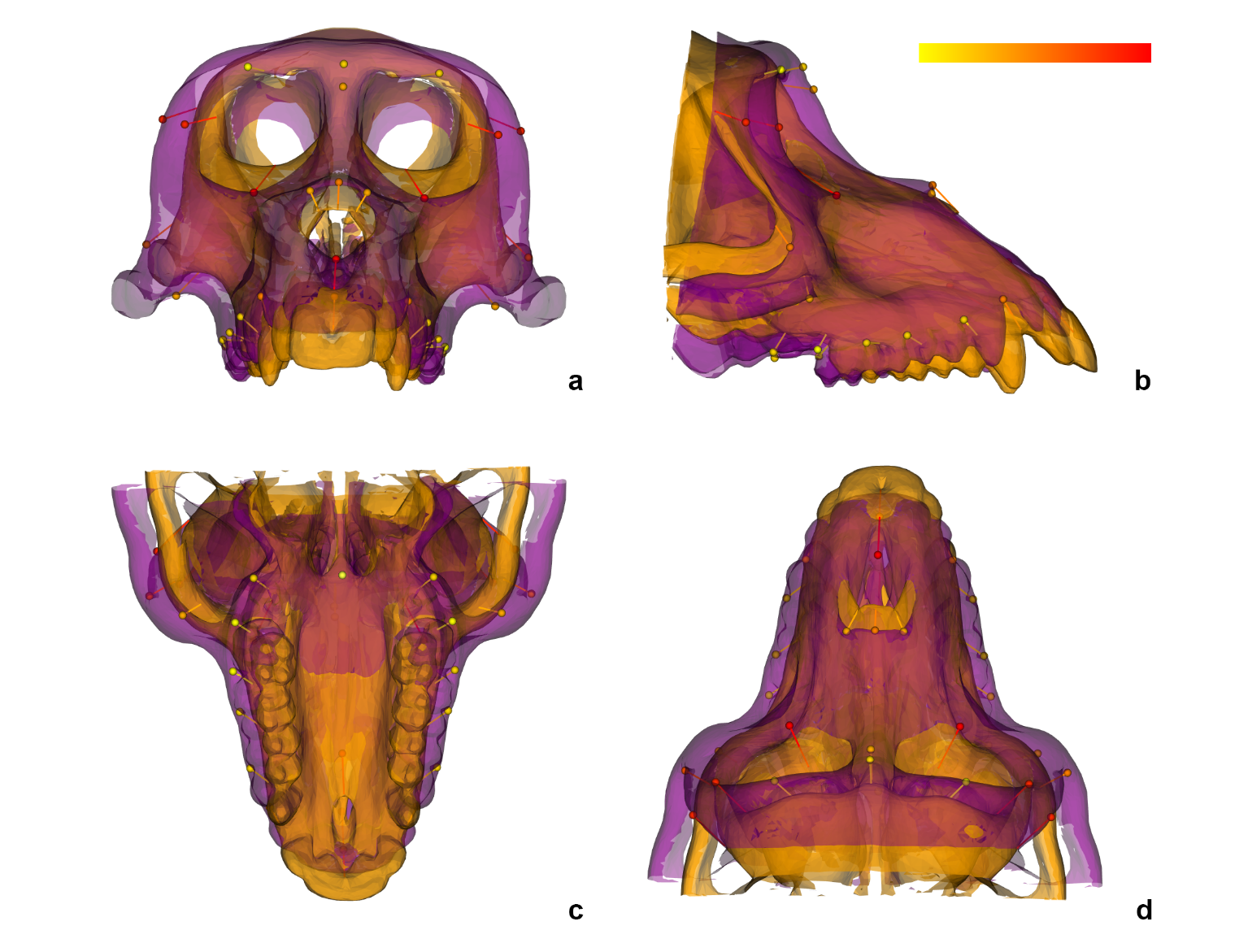


Figure S17**.** Shape changes associated with static allometry (Fig. 5 in the main text). Purple meshes depict the predicted shape in the minimum of regression scores axis, and orange the maximum in a) frontal, b) right lateral, c) ventral, and d) dorsal views. Overlaid are the respective landmarks with the vectors of change. The color scale depicts the magnitude of variation for each landmark.


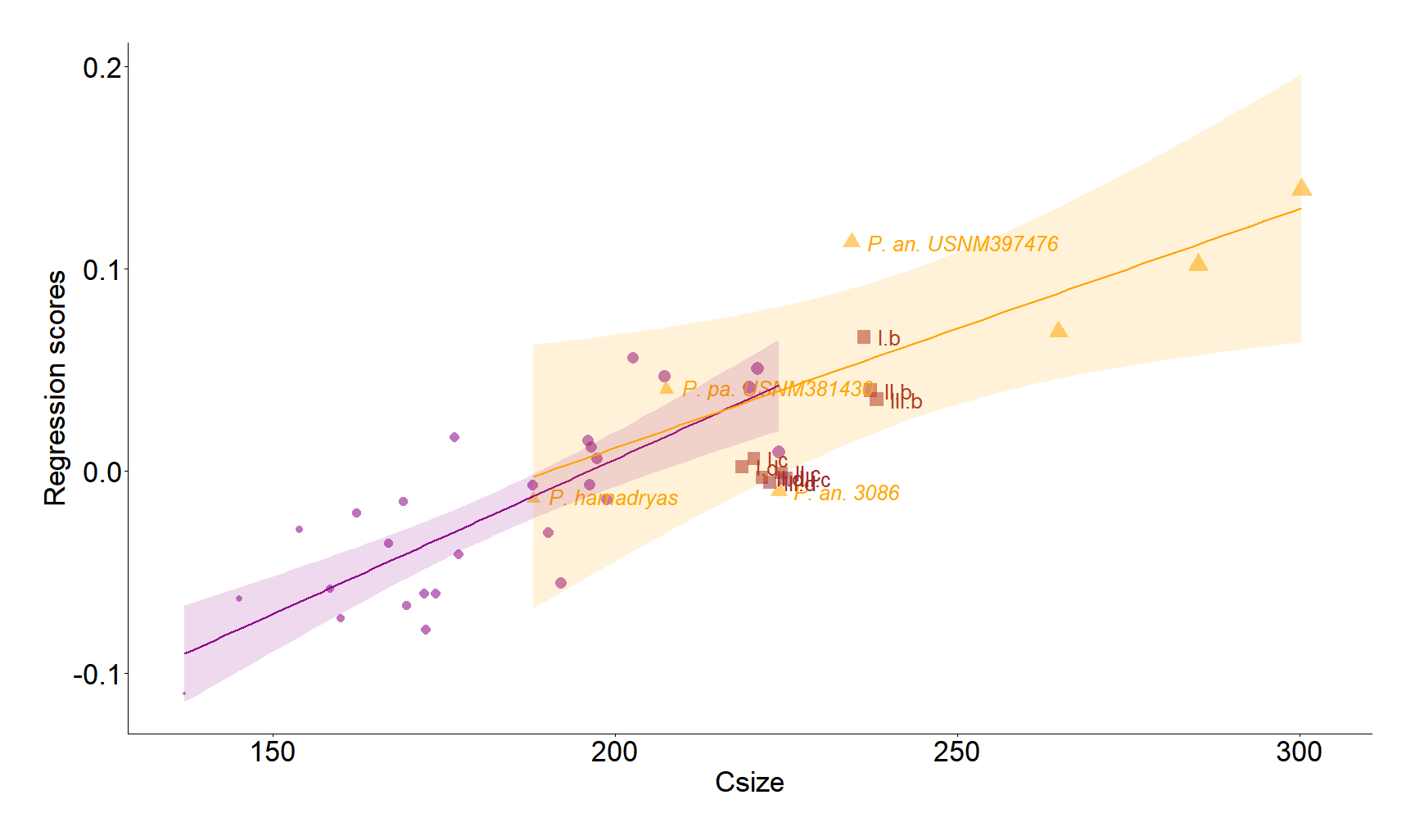


Figure S18**.** Shape scores of the regression with size and genus as predictors, plotted against centroid size. In purple are *Macaca* species, in orange *Papio*, and in brown *Paradolichopithecus* models. Centroid size differences are graphically displayed with the symbol’s size. The purple and orange solid lines are the *Macaca* and *Papio* individual regression lines respectively. Around the lines is the 95% confidence interval area in purple and orange for *Macaca* and *Papio* respectively. Centroid size differences are graphically displayed with the symbol’s size. *Paradolichopithecus* abbreviations follow the Fig. 1 of the main text numeration; letters, b, c, d, correspond to the virtual reconstruction protocol and Latin numbers I, II, III correspond to the template upon which these protocols were applied. Abbreviations in the rest of the comparative material have been omitted. Subadult and/ or female *Papio* individuals are displayed with their abbreviation and catalogue number.


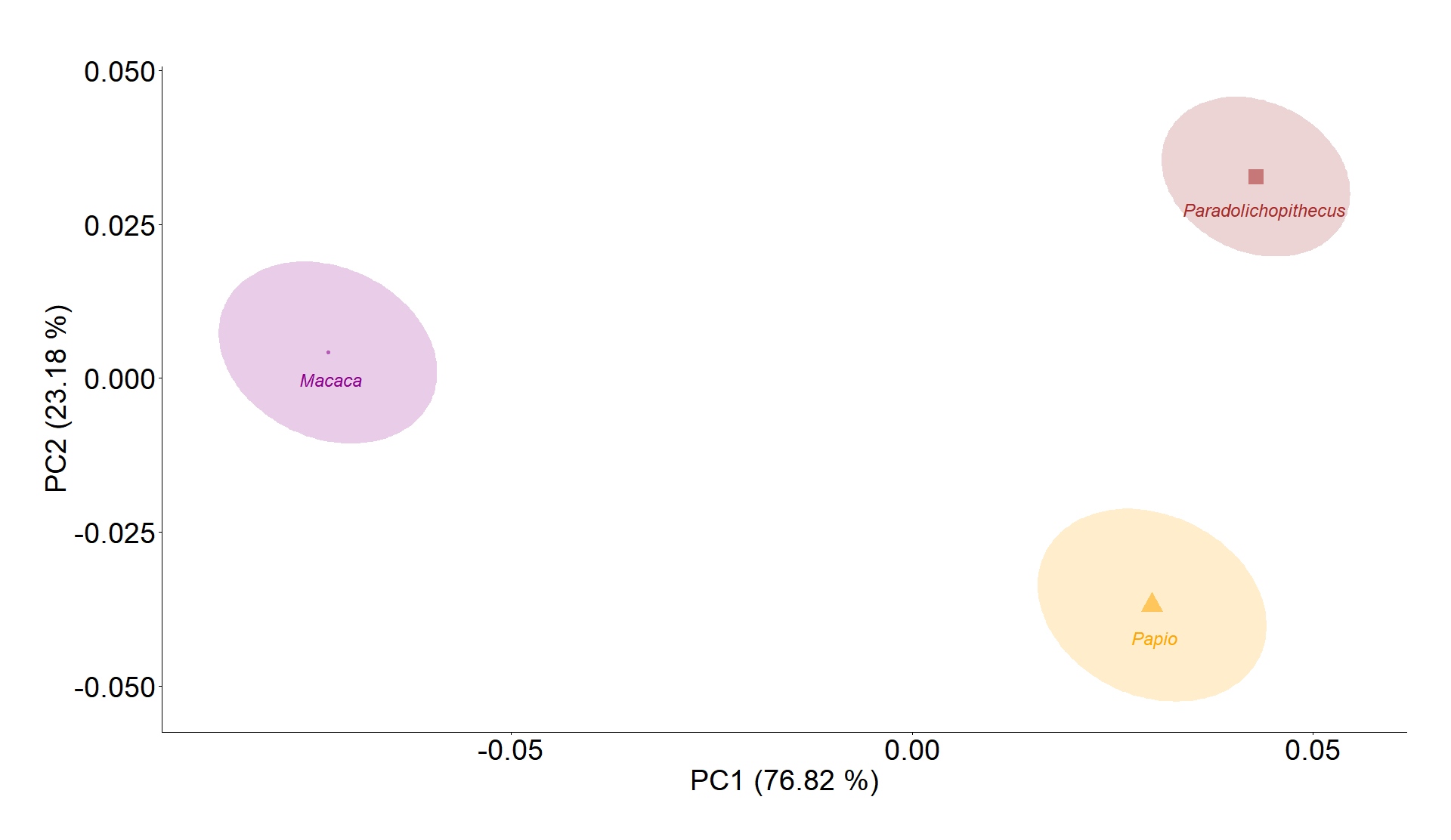


Figure S19. Shape PCA of the predicted mean shapes per genus from the common allometry model while keeping the centroid size constant at the mean value of the comparative sample. Halos depict the 95% confidence ellipses. Genus mean centroid size differences are graphically displayed with the symbol’s size.


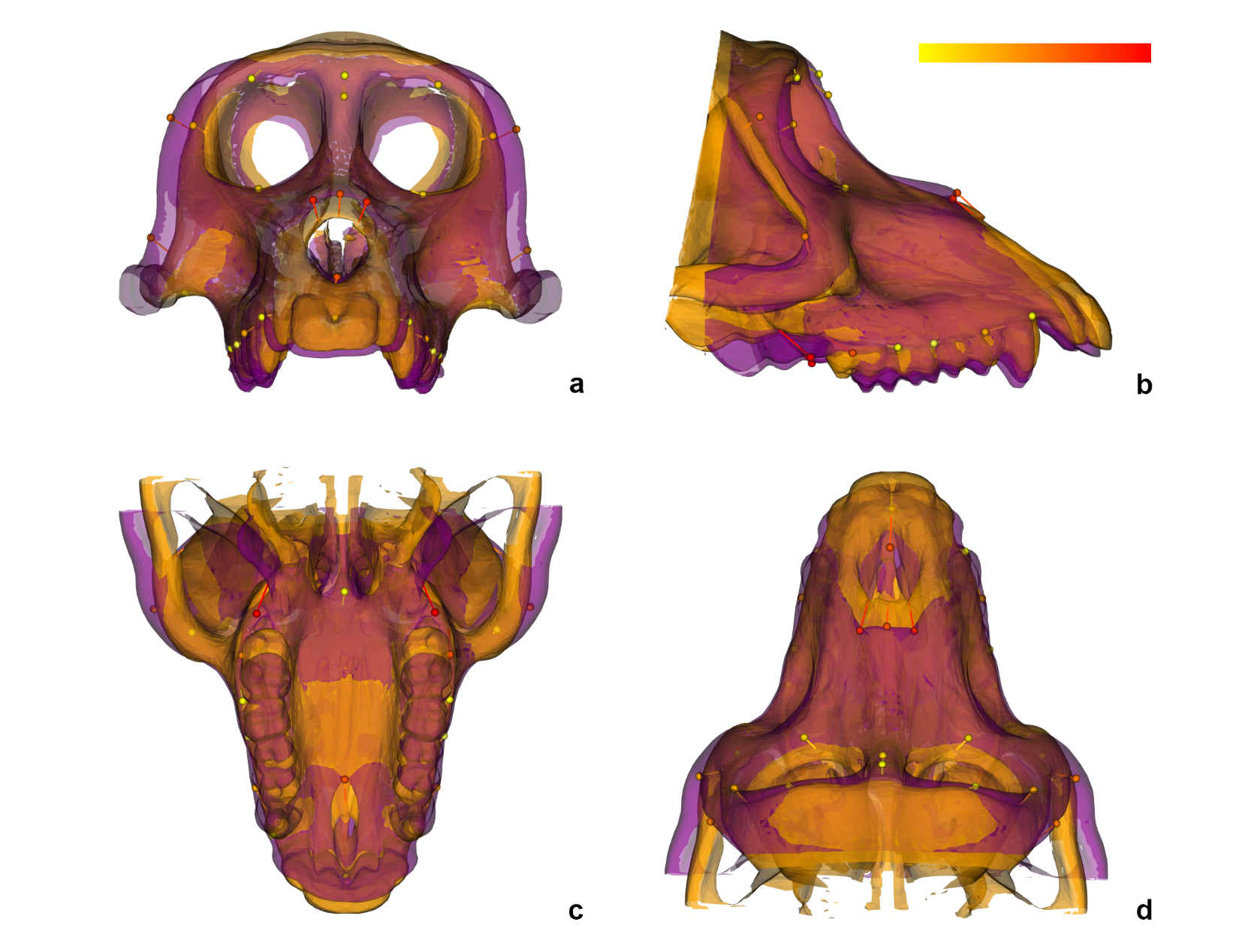


Figure S20**.** Shape changes along the PC1 axis of the predicted shapes for the three genera, when keeping size constant. Purple mesh depicts the predicted shape in the minimum of PC1 axis and orange the maximum in a) frontal, b) right lateral, c) ventral, and d) dorsal views. Overlaid are the respective landmarks with the vectors of change. The color scale depicts the magnitude of variation for each landmark.


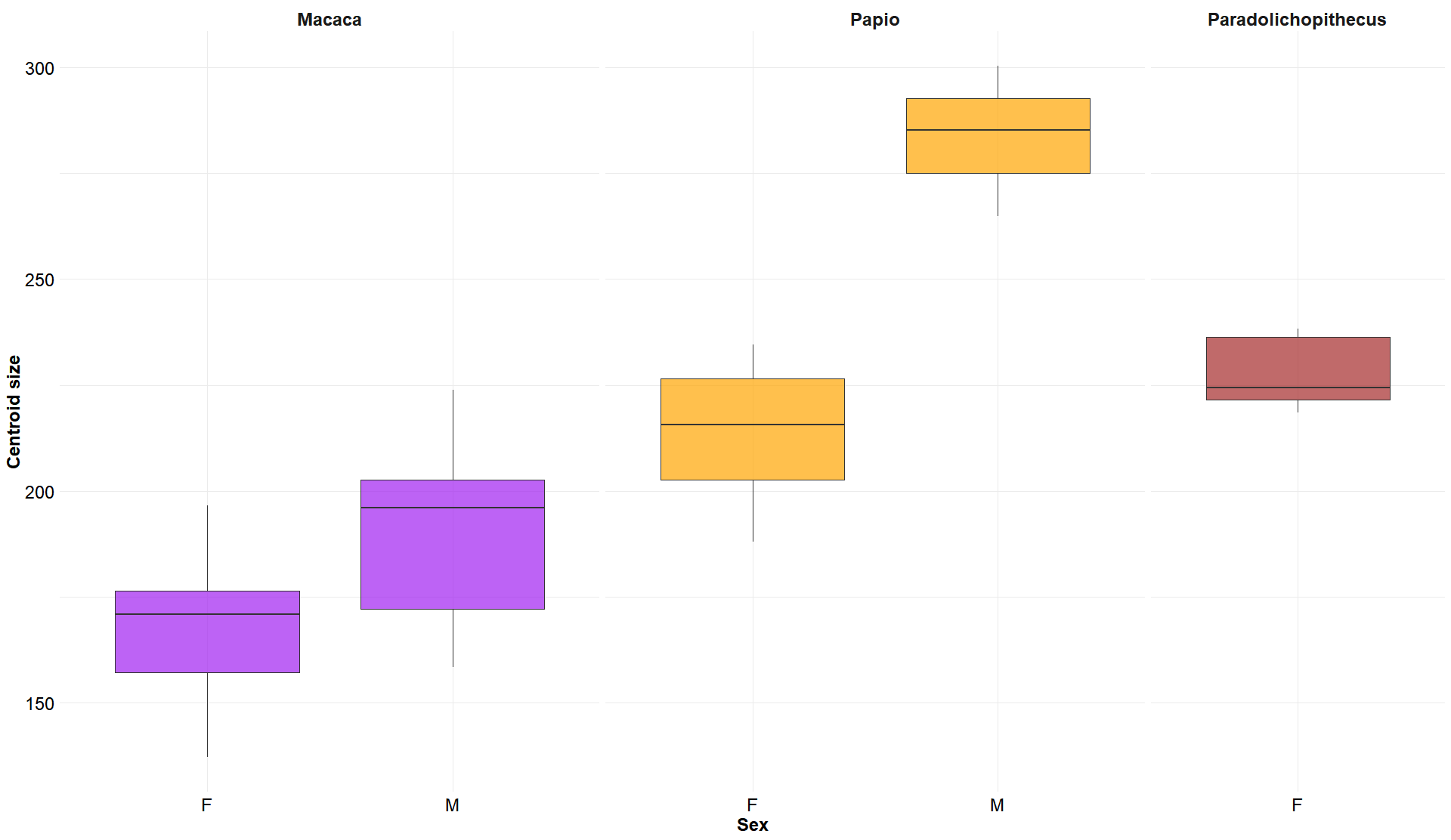


Figure S21**.** Boxplots of centroid size distributions per sex for *Macaca* (in purple), *Papio* (in orange) and *Paradolichopithecus* reconstructions (in brown). **F** denotes females and **M** denotes males.

# Supplementary Tables

Table S1**.** Specimens used in this study.

| ***Species*** | **Catalog Number** | **^a^Macaques and baboons sp. groups** | **Repository** | **Sex** | **^c^Dental score** | **Morph. Media ID/ PRICT No.** | **^e^Resolution** | **Abbreviation** |
| --- | --- | --- | --- | --- | --- | --- | --- | --- |
| *Macaca arctoides* | USNM 111966 | Sinica | NMNH | Female | 5 | Media 000102688 | 0.55 | *M. arctoides* |
| *Macaca arctoides* | USNM 256825 | Sinica | NMNH | Male | 5 | *Smithsonian Collection* | 0.66 | *M. arctoides* |
| *Macaca assamensis* | USNM 15255 | Sinica | NMNH | Female | 5 | *Smithsonian Collection* | 0.54 | *M. assamensis* |
| *Macaca cyclopis* | USNM 296795 | Fascicularis | NMNH | Male | 5 | Media 000103088 | 0.66 | *M. cyclopis* |
| *Macaca fascicularis* | USNM 573504 | Fascicularis | NMNH | Male | 5 | *Smithsonian Collection* | 0.66 | *M. fascicularis* |
| *Macaca fascicularis* | USNM 317191 | Fascicularis | NMNH | Male | 5 | Media 000103157 | 0.62 | *M. fascicularis* |
| *Macaca fascicularis* | USNM 121511 | Fascicularis | NMNH | Male | 5 | Media 000102652 | 0.67 | *M. fascicularis* |
| *Macaca fascicularis* | USNM 256072 | Fascicularis | NMNH | Female | 5 | *Smithsonian Collection* | 0.59 | *M. fascicularis* |
| *Macaca nemestrina* | USNM 241022 | Silenus | NMNH | Male | 5 | Media 000103281 | 0.75 | *M. fascicularis* |
| *Macaca nemestrina^d^* | USNM 114502 | Silenus | NMNH | Female | 5 | Media 000102610 | 0.53 | *M. nemestrina* |
| *Macaca nemestrina* | USNM 123144 | Silenus | NMNH | Male | 5 | *Smithsonian Collection* | 0.73 | *M. nemestrina* |
| *Macaca nemestrina* | USNM 154367 | Silenus | NMNH | Male | 5 | Media 000102461 | 0.58 | *M. nemestrina* |
| *Macaca nemestrina* | USNM 399506 | Silenus | NMNH | Female ? | 5 | Media 000103201 | 0.53 | *M. nemestrina* |
| *Macaca nemestrina x mulata* | USNM 396929 | Silenus | NMNH | Female | 5 | *Smithsonian Collection* | 0.72 | *M. nemestrina* |
| *Macaca radiata* | USNM 398463 | Sinica | NMNH | Female | 5 | Media 000102458 | 0.49 | *M. radiata* |
| *Macaca radiata^d^* | M-163078 | Sinica | AMNH | Male | 5 | Media 000020070 | 0.3 | *M. radiata* |
| *Macaca siberu* | USNM 546835 | Silenus | NMNH | Male | 5 | *Smithsonian Collection* | 0.73 | *M. siberu* |
| *Macaca sinica* | USNM 15259 | Sinica | NMNH | Male | 5 | Media 000103633 | 0.63 | *M. sinica* |
| *Macaca sinica* | USNM 271190 | Sinica | NMNH | Female | 5 | Media 000102305 | 0.42 | *M. sinica* |
| *Macaca sylvanus* | USNM 255979 | Sylvanus | NMNH | Male | 5 | Media 000103248 | 0.57 | *M. sylvanus* |
| *^*1^Macaca sylvanus* | USNM 476782 | Sylvanus | NMNH | Female | 5 | *Smithsonian Collection* | 0.62 | *M. sylvanus* |
| *Macaca sylvanus^d^* | 1392 | Sylvanus | Japan Monkey Center | Male | 5 | 1152 | 0.23 | *M. sylvanus* |
| *Macaca sylvanus* | 6330 | Sylvanus | Japan Monkey Center | Male | 5 | 1138 | 0.23 | *M. sylvanus* |
| *^*2^Macaca sylvanus* | I | Sylvanus | iPHEP, Un. of Poitiers | Male | 5 | I | 0.19 | *M. sylvanus* |
| *Macaca sylvanus^d^* | USNM 476780 | Sylvanus | NMNH | Male | 5 | Media 000020359 | 0.49 | *M. sylvanus* |
| *Macaca thibetana* | USNM 241162 | Sinica | NMNH | Female | 5 | Media 000102867 | 0.54 | *M. thibetana* |
| *Macaca thibetana^d^* | USNM 241163 | Sinica | NMNH | Male | 5 | *Smithsonian Collection* | 0.58 | *M. thibetana* |
| *^*1^Mandrillus sphinx* | 3091 | Mandrills | Primate Research Institute, Kyoto Uni | Female | 4->5 | 70 | 0.22 | *-* |
| *^*1^Papio anubis* | 3086 | Guinea | Primate Research Institute, Kyoto Uni | Female | 4 | 733 | 0.29 | *P. anubis* |
| *Papio anubis^b^* | 1626 | Guinea | Primate Research Institute, Kyoto Uni | Male | 5 | 351 | 0.46 | *P. anubis* |
| *Papio anubis^b^* | USNM 162899 | Guinea | NMNH | Male | 5 | Media 000103139 | 0.62 | *P. anubis* |
| *^*2^Papio anubis^b,d^* | USNM 397476 | Guinea | NMNH | Female | 5 | Media 000102524 | 0.6 | *P. anubis* |
| *Papio hamadryas^b^* | USNM A 49732 | Hamadryas | NMNH | ? | 4 | Media 000103492 | 0.61 | *P. hamadryas* |
| *Papio papio^b,d^* | USNM 378669 | Guinea | NMNH | Male | 5 | Media 000103437 | 0.92 | *P. papio* |
| *Papio papio^b^* | USNM 381430 | Guinea | NMNH | Female | 5 | Media 000102429 | 0.6 | *P. papio* |
| ***^*2^Paradolichopithecus* aff. *arvernensis*** | **DFN3-150** |  | **LGPUT** | **Female** | **4** | DFN3-150 | 0.27 | *-* |

^*1^Used in Amano et al. (2022) as reference crania for the restoration.

^*2^Used in error calculation. *Par.* aff. *arvernensis* ‘wideNasal’ reconstruction was used instead of the ‘original’ model.

^a^Based on Delson, 1980 (in[[7](#_ENREF_7),[8](#_ENREF_8)]).

^b^Taxonomy follows a ‘phylogenetic consensus’ essence[[9-11](#_ENREF_9)].

^c^After[[12](#_ENREF_12)].

^d^Specimens that have been corrected for various structural deformities via mirroring counterparts or filling mesh holes via a convex hull application.

^e^Resolution refers to the mean edge length (in mm) of the single-layer meshes corresponding to the viscerocranium of each species. DFN3-150 resolution is the same for all subsequent reconstructions. Rvcg::vcgMeshres() function[[4](#_ENREF_4)] was used for the calculations.

Table S2**.** Complete list of anatomical landmarks used in the study. Not all landmarks are used in all stages.

| **#** | **Name** | **Position** | **Reference** | **Symbol** | **Definition** | **Type** |
| --- | --- | --- | --- | --- | --- | --- |
| 1 | ^*2^Glabella^c,d,e^ | midline | Frost et al., 2003 | GL | most forward projected point of the frontal bone | 2 |
| 2 | ^*2, 4^Nasion^c,d,e^ | midline | Frost et al., 2003 | NA | point of the fronto-nasal suture | 1 |
| 3 | Rhinion^c,d,e^ | midline | Frost et al., 2003 | RH | most anterior point of the nasal | 1 |
| 4 | ^*2, 4^Nasospinale^c,d,e^ | midline | Frost et al., 2003 | NS | most inferior point of the piriform aperture | 2 |
| 5 | ^*6^Prosthion^c,d,e^ | midline | Frost et al., 2003 | PR | anteroinferior point on projection of premaxilla between central incisors | 2 |
| 6 | Incisivion^c,d,e^ | midline | Frost et al., 2003 | IV | most posterior point of the incisive foramen | 2 |
| 7 | Staphylion^c,d,e^ | midline | Frost et al., 2003 | ST | point located on the tangent to the posterior notches of the horizontal plate of the palatine bone | 1 |
| 8 | ^*5^Frontomalare orbitale^a,b,c,d,e^ | bilateral | Frost et al., 2003 | FMO | point where the frontozygomatic suture cross the inner orbital rim | 2 |
| 9 | ^*5^Frontomalare temporale^a,b,c,d,e^ | bilateral | Frost et al., 2003 | FMT | point where the frontozygomatic suture crosses lateral edge of zygoma | 3 |
| 10 | ^*5^Zygo-max superior^a,b,c,d,e^ | bilateral | Frost et al., 2003 | ZMU | anteriormost point of the zygomaticomaxillary suture on the orbital rim | 2 |
| 11 | ^*5^Zygo-max inferior^d,e^ | bilateral | Nishimura et al.,2019 | ZMI | antero-inferior point of zygomaticomaxillary suture | 2 |
| 12 | Nasal Premaxilla Margin^a,b,c,d,e^ | bilateral | Nishimura et al., 2019 | NPM | Meeting point of nasal and premaxilla on the margin of piriform aperture | 2 |
| 13 | Maximum Zygomatic Arch^a,b,c,d,e^ | bilateral | Nishimura et al., 2019 | CZA | maximum curvature of anterior upper margin of the zygomatic arch | 2 |
| 14 | Mid-Torus inferior^a,c,d,e^ | bilateral | Frost et al., 2003 | MTI | point on inferior margin of supraorbital torus roughly at the middle of the orbital rim | 2 |
| 15 | Anterior Canine Alveolus^a,b,c,d,e^ | bilateral | Nishimura et al., 2019 | ACA | anteriormost point of canine alveolus | 2 |
| 16 | Mesial P3^a,b,c,d,e^ | bilateral | Frost et al., 2003 | MP3 | most mesial point on P3 alveolus projected at the alveolar margin | 2 |
| 17 | P4-M1 Contact^a,b,c,d,e^ | bilateral | Nishimura et al., 2019 | MM1 | most mesial point on M1 alveolus projected at the alveolar margin | 2 |
| 18 | M1-M2 Contact^a,b,c,d,e^ | bilateral | Frost et al., 2003 | M12 | most mesial point on M2 alveolus projected at the alveolar margin | 2 |
| 19 | Mesial M3^a,b,c,d,e^ | bilateral | Nishimura et al., 2019 | MM3 | most mesial point on M3 alveolus projected at the alveolar margin | 2 |
| 20 | Distal M3^a,b,c,d,e^ | bilateral | Frost et al., 2003 | M3D | distal midpoint projected lateraly onto alveolar margin | 2 |
| 21 | Inferior point P3-P4^a,c^ | bilateral | Amano et al., 2022 | 72 | most inferior point on the lingual surface of the maxilla between P3 and P4 | 2 |
| 22 | Inferior point P4-M1^a,c^ | bilateral | Amano et al., 2022 | 73 | most inferior point on the lingual surface of the maxilla between P4 and M1 | 2 |
| 23 | Inferior point M1-M2^1,3^ | bilateral | Amano et al., 2022 | 74 | most inferior point on the lingual surface of the maxilla between M1 and M2 | 2 |
| 24 | Inferior point M2-M3^a,c^ | bilateral | Amano et al., 2022 | 75 | most inferior point on the lingual surface of the maxilla between M2 and M3 | 2 |
| 25 | ^*3^Buccal cusp P3^c^ | bilateral | Amano et al., 2022 | 78 | most inferior point on the buccal cusp of P3 | 3 |
| 26 | Buccal cusp P4^c^ | bilateral | Amano et al., 2022 | 79 | most inferior point on the buccal cusp of P4 | 3 |
| 27 | Distobuccal M1^c^ | bilateral | Amano et al., 2022 | 80 | most inferior point on the distobuccal cusp of M1 | 3 |
| 28 | Mesiobuccal M1^c^ | bilateral | Amano et al., 2022 | 81 | most inferior point on the mesiobuccal cusp of M1 | 3 |
| 29 | Distobuccal M2^c^ | bilateral | Amano et al., 2022 | 82 | most inferior point on the distobuccal cusp of M2 | 3 |
| 30 | Mesiobuccal M2^c^ | bilateral | Amano et al., 2022 | 83 | most inferior point on the mesiobuccal cusp of M2 | 3 |
| 31 | ^*3^Lingual cusp P3^c^ | bilateral | Amano et al., 2022 | 87 | most inferior point on the lingual cusp of P3 | 3 |
| 32 | Lingual cusp P4^c^ | bilateral | Amano et al., 2022 | 88 | most inferior point on the lingual cusp of P4 | 3 |
| 33 | Distolingual M1^c^ | bilateral | Amano et al., 2022 | 89 | most inferior point on the distolingual cusp of M1 | 3 |
| 34 | Mesiolingual M1^c^ | bilateral | Amano et al., 2022 | 90 | most inferior point on the mesiolingual cusp of M1 | 3 |
| 35 | Distolingual M2^c^ | bilateral | Amano et al., 2022 | 91 | most inferior point on the distolingual cusp of M2 | 3 |
| 36 | Mesiolingual M2^c^ | bilateral | Amano et al., 2022 | 92 | most inferior point on the mesiolingual cusp of M2 | 3 |
| 37 | *^1^End of piriform appert.^a,b,c^ | bilateral | present study | ePA | point on the distal end of the piriform aperture | *fake* |

^a^for Schlager et al. (2018) protocol[[2](#_ENREF_2)]

^b^for surface registration protocol

^c^for Amano et al. (2022) protocol[[3](#_ENREF_3)]

^d^analysis for the repeatability of landmarks

^e^for the statistical analysis

^*1^Used in the equidistance of the piriform aperture semilandmarks algorithm for the DFN3-150 models that lack nasospinale. In subsequent steps, it has been deleted.

^*2^glabella and nasion landmarks were calculated via a TPS algorithm to apply the Amano et al. (2022) protocol[[3](#_ENREF_3)] in the ‘original’ DFN3-150 model. Additionally, to reconstruct ‘wideNasal’ and ‘widePalate’, nasospinale was also interpolated via a TPS routine.

^*3^Buccal and lingual cusp of P3 in the *Mandrillus sphinx* PRICT70 specimen used in the Amano et al. (2022) restoration protocol[[3](#_ENREF_3)] were calculated via a tps algorithm from the remaining two reference specimens.

^*4^prosthion was intrerpolated for the statistical analysis via a TPS algorithm for the ‘original’ template. Additionally, nasospinale was also interpolated for the ‘wideNasal’ and ‘widePalate’ tempates.

^*5^Anatomical landmarks (Type I and II; see[[13](#_ENREF_13)]) that were treated as semilandmarks allowed to slide during the pre-analysis step.

Table S3**.** List of curves sampled on the specimens. Values on the right represent the number of landmarks sampled for each stage of the present study. ID index corresponds to Figure S5.

| ID | Name | Schlager et al. (2018)[[2](#_ENREF_2)] | Surface Registration | Amano et al. (2022)[[3](#_ENREF_3)] |
| --- | --- | --- | --- | --- |
| *I* | Orbital margin at the zygomatic | 10 | 10 | 10 |
| *II* | Posterior frontal process of the zygomatic | 13 | 13 | 13 |
| *III* | Lateral supraobital margin | 9 |  | 9 |
| *IV* | Mesial orbital margin |  |  | 18 |
| *V* | Piriform apperture | 12 | 12 | 12 |
| *VI* | Nasal segment (Midsagittal) |  |  |  |

Table S4**.** Absolute vertex to surface (i) and vertex to vertex (ii) displacement^a^ between the respected reference specimen meshes after transforming them for the reflected relabeling and mirroring of landmarks applications used in the Amano et al. (2022) restoration protocol[[3](#_ENREF_3)]. Measurements are in mm.

| ***i.*** | **min surface displacement** | **max surface displacement** | **Sd surface displacement** |
| --- | --- | --- | --- |
| *Macaca sylvanus* USNM479782 | 0 | 4.42 | 0.5 |
| *Mandrillus sphinx* PRICT70 | 0 | 0.82 | 0.13 |
| *Papio anubis* PRICT733 | 0 | 1.47 | 0.28 |
| ***ii.*** | **min vertex displacement** | **max vertex displacement** | **Sd vertex displacement** |
| *Macaca sylvanus* USNM479782 | 0 | 5.25 | 1.22 |
| *Mandrillus sphinx* PRICT70 | 0 | 1.474 | 0.27 |
| *Papio anubis* PRICT733 | 0 | 2.54 | 0.41 |

^a^After[[2](#_ENREF_2)]

Table S5**.** Absolute vertex to surface (i) and vertex to vertex (ii) displacement^a^ of the respected reference meshes after transforming for the reflected relabeling (RR) and mirroring of landmarks (Mirror) applications used in the Amano et al. (2022) restoration protocol[[3](#_ENREF_3)], versus their original meshes. Measurements are in mm.

| ***RR*** | ***i.*** | **min sur. displacement** | **max sur. displacement** | **Sd sur. displacement** |
| --- | --- | --- | --- | --- |
|  | *Macaca sylvanus* USNM479782 | 0 | 3.6 | 0.36 |
|  | *Mandrillus sphinx* PRICT70 | 0 | 0.828 | 0.11 |
|  | *Papio anubis* PRICT733 | 0 | 1.082 | 0.13 |
|  | ***ii.*** | **min vertex displacement** | **max vertex displacement** | **Sd vertex displacement** |
|  | *Macaca sylvanus* USNM479782 | 0 | 5.71 | 0.89 |
|  | *Mandrillus sphinx* PRICT70 | 0 | 1.25 | 0.21 |
|  | *Papio anubis* PRICT733 | 0 | 2.071 | 0.23 |
| ***Mirror*** | ***i.*** | **min sur. displacement** | **max sur. displacement** | **Sd sur. displacement** |
|  | *Macaca sylvanus* USNM479782 | 0 | 5.785 | 0.63 |
|  | *Mandrillus sphinx* PRICT70 | 0 | 1.23 | 0.16 |
|  | *Papio anubis* PRICT733 | 0 | 1.77 | 0.32 |
|  | ***ii.*** | **min vertex displacement** | **max vertex displacement** | **Sd vertex displacement** |
|  | *Macaca sylvanus* USNM479782 | 0 | 11.298 | 1.67 |
|  | *Mandrillus sphinx* PRICT70 | 0 | 2.155 | 0.32 |
|  | *Papio anubis* PRICT733 | 0 | 3.38 | 0.41 |

^a^After[[2](#_ENREF_2)]

Table S6**.** The precision of raw coordinates of ‘anchor’ landmarks for the surface registration for three sampling rounds against their Standard deviation (Sd). Measurements are portrayed as Euclidean distance (ED) in mm and Percentage difference (Percentage %).

|  |  | ***ED*** | | | ***Percentage %*** | | |
| --- | --- | --- | --- | --- | --- | --- | --- |
|  |  | **min** | **max** | **Sd** | **min** | **max** | **Sd** |
| *left* | Configuration 1 | 0.0009 | 0.79 | 0.11 | 0.002 | 2.5 | 0.39 |
|  | Configuration 2 | 0.0008 | 0.42 | 0.07 | 0.002 | 1.32 | 0.28 |
|  | Configuration 3 | 0.0003 | 0.38 | 0.07 | 0.002 | 2.03 | 0.33 |
| *right* |  | **min** | **max** | **Sd** | **min** | **max** | **Sd** |
|  | Configuration 1 | 0.001 | 0.33 | 0.07 | 0.007 | 1.43 | 0.3 |
|  | Configuration 2 | 0.0006 | 0.22 | 0.06 | 0.003 | 1.08 | 0.23 |
|  | Configuration 3 | 0.001 | 0.23 | 0.06 | 0.003 | 0.71 | 0.24 |

Table S7**.** Table describing for each configuration’s registered surface, the percentage number of vertices (99,183 total) whose Euclidean Distance from their respected target surface (vertex to surface calculation) is below three predefined thresholds (Percentage of vertices%), followed by their standard deviation (Sd) of Euclidean Distance (ED) and the Riemannian Distance^a^ correlation matrix between all complete resulting configurations of landmarks (RD). RD depicts the proximity of their shapes following a <0.05 threshold[[14](#_ENREF_14)]for high consistency. ED measurements are in mm.

|  | **Percentage of vertices%** | | | **ED** | **RD** | |  |
| --- | --- | --- | --- | --- | --- | --- | --- |
|  | **<2.5μm** | **<0.1mm** | **<0.25mm** | **Sd** | Configuration 1 | Configuration 2 |  |
| Configuration 1 | | 18.1 | 88.28 | 96.56 | 0.08 | - | - |
| Configuration 2 | | 18.07 | 88.00 | 96.61 | 0.07 | 0.017 | - |
| Configuration 3 | | 18.38 | 88.47 | 96.80 | 0.07 | 0.013 | 0.011 |

^a^The geodesic-Riemannian distance between two shapes on a pre-shape space, also referred to as Procrustes Distance (see[[15](#_ENREF_15)]).

Table S8**.** Type II Sum of Squares two-way Procrustes ANOVA table for the symmetric component of the repeated configurations.

|  | ***Df*** | ***SS*** | ***MS*** | ***Rsq*** | ***F*** | ***Z*** | ***Pr*** |
| --- | --- | --- | --- | --- | --- | --- | --- |
| *Repetitions* | 3 | 0.000684 | 0.000228 | 0.00361 | 0.4917 | -1.2044 | 0.88 |
| *Species* | 2 | 0.185854 | 0.092927 | 0.98169 | 200.32 | 4.5715 | **0.0041** |
| *Residuals* | 6 | 0.002783 | 0.000464 | 0.0147 |  |  |  |
| *Total* | 11 | 0.189321 |  |  |  |  |  |

Table S9**.** Type I Sum of Squares blocked perMANOVA.

|  | ***Df*** | ***Sum sq*** | ***R sq*** | ***F*** | ***Pr(>F)*** |
| --- | --- | --- | --- | --- | --- |
| *Model* | 3 | 0.000684 | 0.00361 | 0.0097 | 0.97 |
| *Residual* | 8 | 0.188637 | 0.99639 |  |  |
| *Total* | 11 | 0.189321 | 1 |  |  |

Table S10**.** Type I Sum of Squares Permutation test for homogeneity of multivariate dispersions for i) Trials and ii) Species groups.

|  |  | ***Df*** | ***Sum sq*** | ***Mean sq*** | ***F*** | ***Perm*** | ***Pr(>F)*** |
| --- | --- | --- | --- | --- | --- | --- | --- |
| **i. Trials** | *Groups* | 3 | 0.0000639 | 0.0000213 | 0.0145 | 10000 | 0.9 |
|  | *Residuals* | 8 | 0.0117642 | 0.0014705 |  |  |  |
| **ii. Species** | *Groups* | 2 | 0.00025015 | 0.00012508 | 0.603 | 10000 | 0.66 |
|  | *Residuals* | 9 | 0.00186621 | 0.000020736 |  |  |  |

Table S11**.** Pairwise Riemannian Distance (RD) between sampling trials for all three specimens used. An acceptance threshold value <0.05 for proximity is used.

|  |  |  |  |  |
| --- | --- | --- | --- | --- |
| *Macaca sylvanus* I | **t_1_** | **t_2_** | **t_3_** | **Mean** |
| **t_1_** |  |  |  | 0.018 |
| **t_2_** | 0.009 |  |  |  |
| **t_3_** | 0.025 | 0.025 |  |  |
| **t_4_** | 0.028 | 0.029 | 0.028 |  |
| *Papio anubis* USNM 397476 |  |  |  |  |
| **t_1_** |  |  |  | 0.027 |
| **t_2_** | 0.012 |  |  |  |
| **t_3_** | 0.014 | 0.014 |  |  |
| **t_4_** | **0.059** | **0.058** | **0.059** |  |
| *Par.* aff. a*rvernensis* DFN3-150 |  |  |  |  |
| **t_1_** |  |  |  | 0.012 |
| **t_2_** | 0.0125 |  |  |  |
| **t_3_** | 0.014 | 0.015 |  |  |
| **t_4_** | 0.018 | 0.019 | 0.016 |  |

Table S12**.** Correlation of centroid size measurements with the first two PC scores (Cumulative variance 74.33%).

|  |  | **PC1** | **PC2** |
| --- | --- | --- | --- |
| ^a^Kendall‘s ***τ*** | coefficient | 0.55 | 0.29 |
|  | p-value | <0.001 | 0.01 |
| Spearman‘s ***ρ*** | coefficient | 0.72 | 0.44 |
|  | p-value | <0.001 | 0.009 |

^a^In the main text, only Kendall‘s ***τ*** is referred.

Table S13**.** Results for the test for homogeneity of multivariate dispersion for the genera variable. The test was conducted on the Euclidean distance matrix of the aligned tangent space coordinates.

|  | ***Df*** | ***Sum sq*** | ***Mean sq*** | ***F*** | ***Pr(>F)*** |
| --- | --- | --- | --- | --- | --- |
| *Groups* | 2 | 0.0001 | 0.0001 | 0.348 | 0.534 |
| *Residual* | 32 | 0.0119 | 0.0003 |  |  |
|  |  |  |  |  |  |

Table S14**.** Various test results for the evaluation of the independence of size from genus level on the comparative material.

| ***Genera*** | ***Test*** | ***Statistic*** | ***pvalue*** |
| --- | --- | --- | --- |
| *Macaca* | Shapiro-Wilk | 0.975 | 0.74 |
| *Papio* | Shapiro-Wilk | 0.960 | 0.82 |
|  | Bartlett's test | 4.027 | 0.04 |
|  | Wilcoxon-Mann-Whitney | -3.38^a^ | <0.001 |
|  | Wilcox effect size (r value) | 0.581 | NA |

^a^Z-score.

Table S15**.** Pairwise test of Homogeneity of Slopes (HOS).

|  | **r** | **angle** | **UCL (95%)** | **Z** | **Pr > angle** |
| --- | --- | --- | --- | --- | --- |
| *Macaca* : *Papio* | 0.81 | 35.48 | 47.49 | 0.55 | 0.29 |
|  |  |  |  |  |  |

Table S16**.** Results from the rarefaction analysis of slopes in the unique and common allometries model (OLS), showing the observed slope and the 95% confidence intervals of the observed p value.

|  | | **p-value** | **Confidence Intervals** |
| --- | --- | --- | --- |
| **Rarefied slopes** | Median | 0.4 |  |
|  | Median CI | **0.04** | 2.50% |
|  |  | 0.85 | 97.50% |
| **NULL rarefied slopes** | Median | 0.69 |  |
|  | Median CI | 0.19 | 2.50% |
|  |  | 0.96 | 97.50% |

Table S17**.** Phylogenetic MANCOVA table for the multivariate regression of species mean tangent space coordinates against their centroid size and genus (macaques: genus *Macaca* and baboons: genus *Papio*).

|  |  | **Df** | **SS** | **MS** | **Rsq** | **F** | **Z** | **Pr(>F)** |
| --- | --- | --- | --- | --- | --- | --- | --- | --- |
| **phyMANCOVA** | Size | 1 | 84.321 | 84.321 | 0.278 | 12.593 | 3.91 | <0.001 |
|  | Genus | 1 | 1.409 | 1.409 | 0.004 | 0.21 | -3.007 | 0.998 |
|  | Size : Genus | 1 | 16.571 | 16.571 | 0.054 | 2.474 | 2.082 | 0.018 |
|  | Residuals | 30 | 200.868 | 7.023 | 0.662 |  |  |  |
|  | Total | 33 | 303.243 |  |  |  |  |  |

Table S18**.** Results from the rarefaction analysis of slopes in the unique and common allometries model for the phylogenetic MANCOVA (GLS), showing the observed slope and the 95% confidence intervals of the differences (in degrees), and the observed p value. The common allometry model is considered the null hypothesis model in the test.

|  | | **p-value** | **Confidence Intervals** |
| --- | --- | --- | --- |
| **Rarefied slopes** | Median | 0.88 |  |
|  | Median CI | 0.08 | 2.50% |
|  |  | 1 | 97.50% |
| **NULL rarefied slopes** | Median | 0.09 |  |
|  | Median CI | 0 | 2.50% |
|  |  | 0.84 | 97.50% |

# References

1 Koutalis, S. *Α virtual reconstruction of the deformed DFN3-150 Early Pleistocene Paradolichopithecus cranium from Dafnero-3, Greece* Master's thesis, Aristotle University of Thessaloniki, (2022).

2 Schlager, S., Profico, A., Di Vincenzo, F. & Manzi, G. Retrodeformation of fossil specimens based on 3D bilateral semi-landmarks: Implementation in the R package "Morpho". *PLoS One* **13**, e0194073 (2018). <https://doi.org/10.1371/journal.pone.0194073>

3 Amano, H., Rae, T. C., Tsoukala, E., Nakatsukasa, M. & Ogihara, N. Computerized restoration of a fossil cranium based on selective elimination of estimated taphonomic deformation. *American Journal of Biological Anthropology* **178**, 448-460 (2022). <https://doi.org/10.1002/ajpa.24493>

4 Schlager, S. *Morpho and Rvcg - Shape Analysis in R*. 217-256 (Academic Press, 2017).

5 Watanabe, A. How many landmarks are enough to characterize shape and size variation? *PLoS One* **13**, e0198341 (2018). <https://doi.org/10.1371/journal.pone.0198341>

6 Arnold, C., Matthews, L. J. & Nunn, C. L. The 10kTrees website: A new online resource for primate phylogeny. *Evolutionary Anthropology: Issues, News, and Reviews* **19**, 114-118 (2010). <https://doi.org/10.1002/evan.20251>

7 Nishimura, T., Morimoto, N. & Ito, T. Shape variation in the facial part of the cranium in macaques and African papionins using geometric morphometrics. *Primates* **60**, 401-419 (2019). <https://doi.org/10.1007/s10329-019-00740-1>

8 Li, J. *et al.* Phylogeny of the macaques (Cercopithecidae: Macaca) based on Alu elements. *Gene* **448**, 242-249 (2009). <https://doi.org/10.1016/j.gene.2009.05.013>

9 Kopp, G. H. *et al.* A Comprehensive Overview of Baboon Phylogenetic History. *Genes (Basel)* **14** (2023). <https://doi.org/10.3390/genes14030614>

10 Zinner, D., Wertheimer, J., Liedigk, R., Groeneveld, L. F. & Roos, C. Baboon phylogeny as inferred from complete mitochondrial genomes. *Am J Phys Anthropol* **150**, 133-140 (2013). <https://doi.org/10.1002/ajpa.22185>

11 Jordan, V. E. *et al.* A computational reconstruction of Papio phylogeny using Alu insertion polymorphisms. *Mob DNA* **9**, 13 (2018). <https://doi.org/10.1186/s13100-018-0118-3>

12 Schillaci, M. A., Froehlich, J. W. & Supriatna, J. Growth and sexual dimorphism in a population of hybrid macaques. *Journal of Zoology* **271**, 328-343 (2006). <https://doi.org/10.1111/j.1469-7998.2006.00208.x>

13 Caple, J. & Stephan, C. N. A standardized nomenclature for craniofacial and facial anthropometry. *Int J Legal Med* **130**, 863-879 (2016). <https://doi.org/10.1007/s00414-015-1292-1>

14 Profico, A. *et al.* The evolution of cranial base and face in Cercopithecoidea and Hominoidea: Modularity and morphological integration. *Am J Primatol* **79** (2017). <https://doi.org/10.1002/ajp.22721>

15 Klingenberg, C. P. Walking on Kendall’s Shape Space: Understanding Shape Spaces and Their Coordinate Systems. *Evolutionary Biology* **47**, 334-352 (2020). <https://doi.org/10.1007/s11692-020-09513-x>
