# Supplementary material for: Virtual reconstruction and analysis of the face of DFN3-150 Paradolichopithecus aff. arvernensis specimen from Dafnero, Greece
Source: Sci Rep. 2026 May 10;16:14703. doi: 10.1038/s41598-026-51595-8 (PMC13158299; doi:10.1038/s41598-026-51595-8)
Supplement: Supplementary file 2 — Supplementary Material 2 [file 41598_2026_51595_MOESM2_ESM.pdf]

Virtual reconstruction and analysis of the face of DFN3-150 *Paradolichopithecus* aff. *arvernensis* specimen from Dafnero, Greece.

Stylianos Koutalis, Carolin Röding, Gildas Merceron, Franck Guy, Dimitris S. Kostopoulos, Katerina Harvati

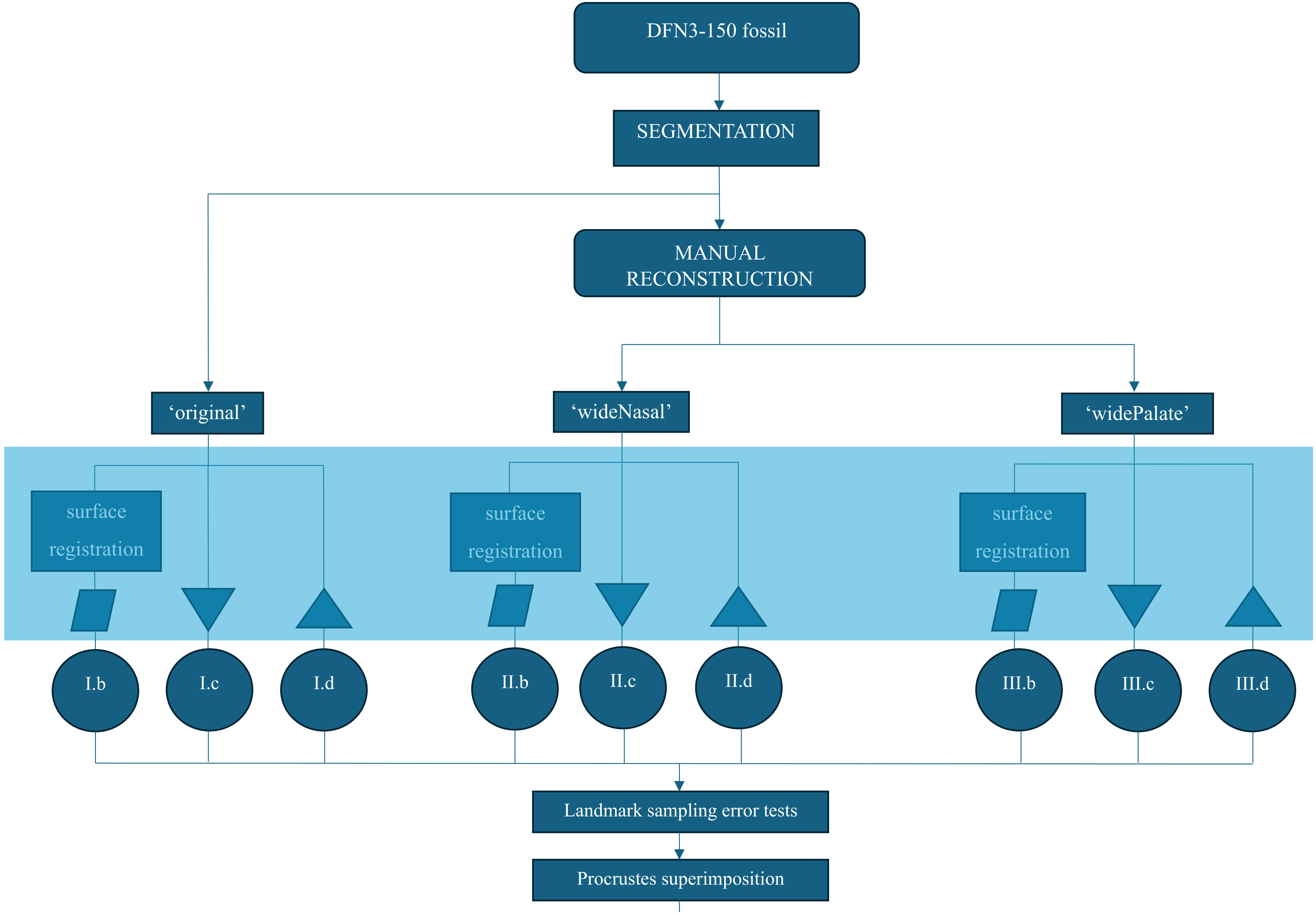

Flowchart 1. Workflow following the initial segmentation of DFN3-150. Deformed rectangle depicts the application Schlager et. al., (2018), reversed triangle is the application of Amano et. al., (2022) with semilandmarks, and triangle the same protocol without semilandmarks. Circles depict the virtually retrodeformed models. Numeration of the models follows Fig. 1 in the main text. Light blue box is the inner procedures preceding the generation of the models, like surface registration.

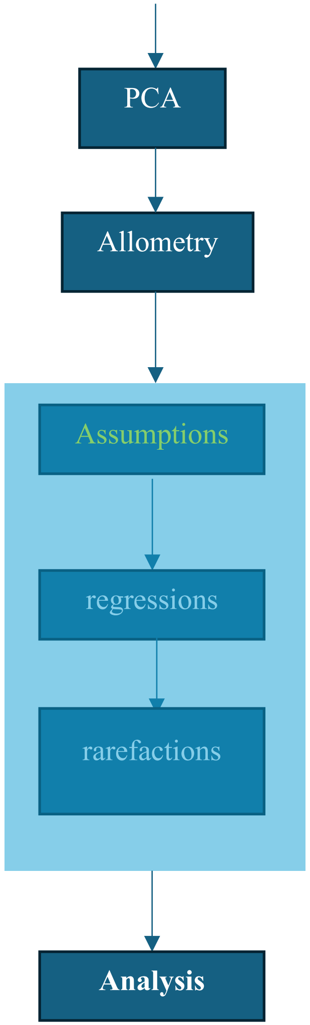

Flowchart 2. The main analyses performed in this study, after *Flowchart 1.* Procrustes registration.

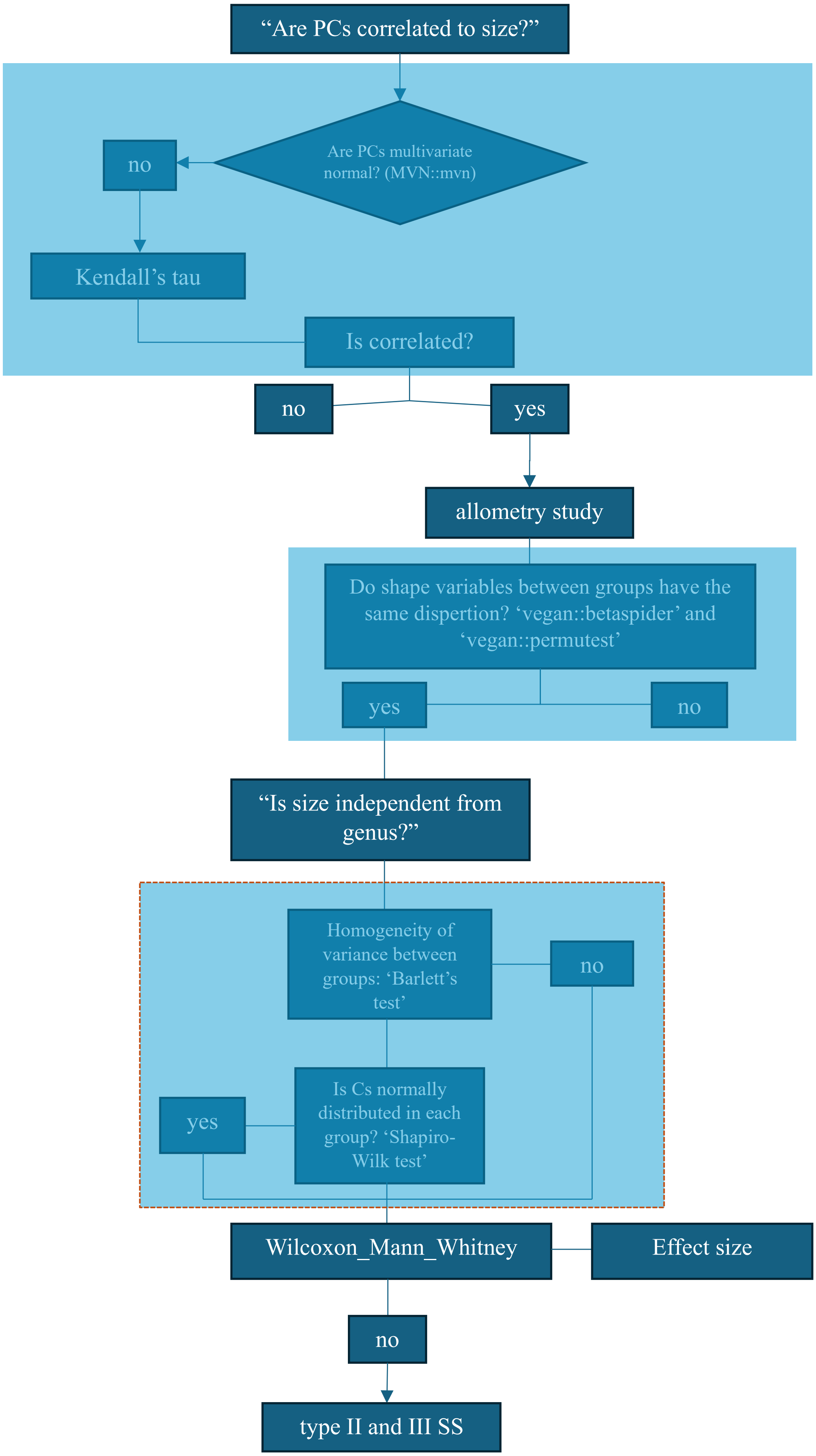

Flowchart 3. Workflow documenting the argumentation that follows the allometric analysis after the initial PCA. Light blue boxes depict inner procedures that address the question above them. Dashed orange box displays an AND logical operator.
